# Multi-omics analysis reveals the metabolic regulators of duodenal low-grade inflammation in a functional dyspepsia model

**DOI:** 10.3389/fimmu.2022.944591

**Published:** 2022-08-24

**Authors:** Shuai Ji, Yanting You, Baizhao Peng, Tianyu Zhong, Yuxiang Kuang, Shasha Li, Lijing Du, Liqian Chen, Xiaomin Sun, Jiaojiao Dai, Suiping Huang, Yuyao Wu, Yanyan Liu

**Affiliations:** ^1^ School of Traditional Chinese Medicine, Southern Medical University, Guangzhou, China; ^2^ The Second Clinical College of Guangzhou University of Chinese Medicine, Guangzhou, China; ^3^ School of Pharmacy, Shanghai Jiao Tong University, Shanghai, China; ^4^ Integrated Hospital of Traditional Chinese Medicine, Southern Medical University, Guangzhou, China

**Keywords:** multi-omics, innate immune, inflammation, endocannabinoid, functional dyspepsia

## Abstract

Several gastrointestinal phenotypes and impairment of duodenal mucosal barrier have been reported in clinical studies in patients with functional dyspepsia (FD). Due to the preferential colonization of the mucosa, intestinal microbes and their metabolites are commonly involved in host metabolism and immune responses. However, there are no studies on the intertwined correlation among multi-level data. For more comprehensive illustrating, a multi-omics analysis focusing on the duodenum was performed in the FD rat model. We found that differential microbiomes in the duodenum were significantly correlated with the biosynthesis of lipopolysaccharide and peptidoglycan. The innate immune response-related genes, which were upregulated in the duodenum, were associated with the TLR2/TLR4-NFκB signaling pathway. More importantly, arachidonyl ethanolamide (anandamide, AEA) and endocannabinoid analogues showed linear relationships with the FD phenotypes. Taken together, multi-level data from microbiome, transcriptome and metabolome reveal that AEA may regulate duodenal low-grade inflammation in FD. These results suggest an important cue of gut microbiome–endocannabinoid system axis in the pathogenesis of FD.

## Introduction

Studies have shown that the prevalence of FD is approximately 16% in the general population, although with potential regional and diagnosis-related variations (1). The principles of treatment with a better biopsychosocial understanding of the gut–brain axis have been highlighted (2). A recent study reported that the vagal gut-brain signaling regulates both the cerebral pain perception and the structural plasticity of FD in a “bottom-up” manner (3); however, this may not have a high clinical translational potential. Clinical data have confirmed that duodenal barrier disruption does exist in the patients with FD (4). The potential damage-associated molecular patterns (DAMPs), but not the pathogens themself, can enter though impaired intestinal barrier, resulting in the host innate immune responses and a low-grade inflammatory condition (5).

The endocannabinoid system, especially the arachidonyl ethanolamide (AEA), was first proposed to be a regulator of energy balance and gastrointestinal load with brainstem-duodenum neural connections (6). Earlier studies have proved that AEA has a crucial role in the physiological regulation of gastric emptying (7). Also, the dysregulation of peripheral AEA is involved in the modulation of small intestinal motility, with a high level in the duodenum (8). However, no studies have investigated whether AEA is involved in the pathophysiological processes of FD.

In this study, we introduce a combined approach including multi-omics data of microbiome, untargeted metabolome and transcriptome to explore the mechanisms underlying FD pathogenesis. Microbial disturbances and predicted metabolic enzymes associated with the biosynthesis of lipopolysaccharide and peptidoglycan are detected in the duodenum. The duodenal protein-coding genes related to host innate immune response are associated with the Toll-like receptor (TLR) signaling and NK-κB-mediated inflammatory signaling pathways. Interestingly, AEA and several endocannabinoid analogues identified in the duodenal with distinct metabolic patterns are significantly correlated with the phenotypes found in FD. These results provide strong evidence that the “gut microbiome–endocannabinoid system axis” in the duodenum is a novel biomarker and therapeutic target for the treatments of functional gastrointestinal disorders.

## Results

### Compromised gastrointestinal motor function and impaired duodenal mucosal barrier were accompanied with reduction of immune organs index in the rat model of FD

**Figure 1:**
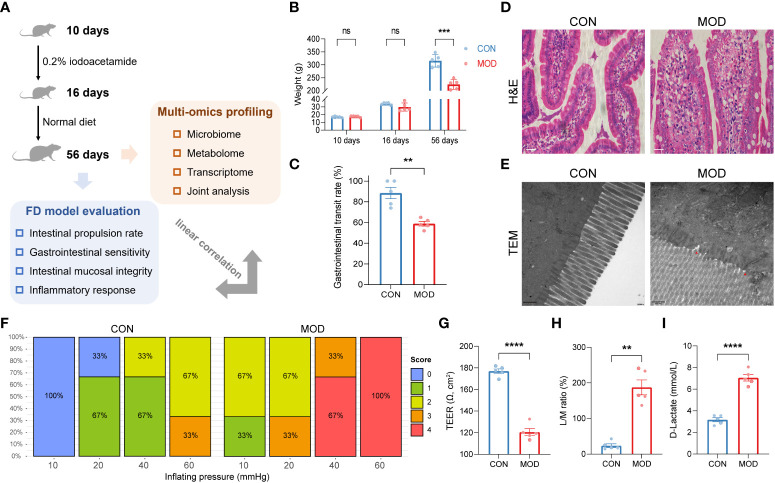
Phenotypes found in the rat model of FD. **(A)** A schematic diagram of the multi-omics analysis. **(B)** Changes in body weights (*** *p* < 0.001 by two-way ANOVA with correction of two-stage linear step-up procedure of Benjamini, Krieger and Yekutieli, ns indicates not significant). **(C)** Gastrointestinal transit rate. **(D)** H&E-stained duodenum. Scale bar, 20 μm. **(E)** TEM analysis of duodenum. Scale bar, 500 nm. Two red triangles indicate incomplete tight junctions. **(F)** Behavioral testing of GD (10 to 60 mmHg) and pain score (0-4). **(G)** TEER of duodenal epithelial barrier (**** *p* < 0.0001 by unpaired t test with Welch’s correction). **(H)** Urine L/M ratio (** *p* < 0.01 by Mann Whitney test). **(I)** Plasma content of D-lactate (**** *p* < 0.0001 by unpaired t test with Welch’s correction). The circles on the bar plots represent individual values with mean ± SEM (bars) (*n* = 3 or 5 rats/group).

The experimental workflow is illustrated in **Figure 1A**. Using the classical method, gavage of iodoacetamide for six consecutive days at young age significantly altered body weight in adult rats (**Figure 1B**). Compared to the control group, the gastrointestinal transit rate was significantly lower in the model group (**Figure 1C**). However, no significant signs of duodenal damage were observed between the groups (**Figure 1D**) but only a small amount of incomplete tight junctions (**Figure 1E**). With further increasing pressure (air injection volume), the model group had higher scores on gastrointestinal sensitivity measures compared with the control group (**Figure 1F**). An additional trans-endothelial electrical resistance (TEER) experiment was conducted to investigate the duodenal barrier phenotypes in detail. A significant reduction of TEER was observed in the model group (**Figure 1G**). As shown in **Figure 1H**, a higher lactulose/mannitol (L/M) ratio in the model group was indicative of increased permeability and impairment of absorption. Similarly, the plasma content of D-lactate was significantly increased in the model group (**Figure 1I**).

**Figure 2:**
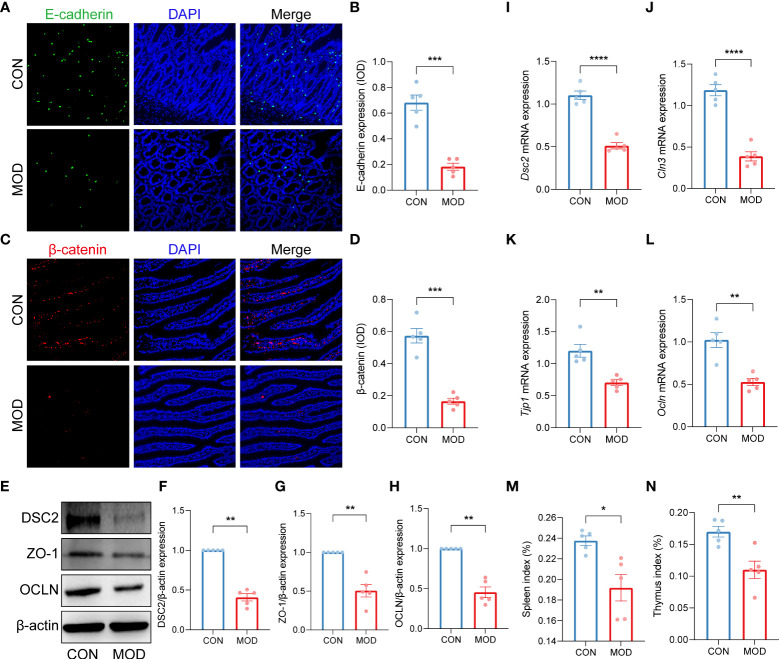
Impaired duodenal mucosal barrier was accompanied with decreased immune organs index. **(A-D)** Duodenal immunofluorescence staining **(A, C)** and the relative expression (IOD) of E-cadherin **(B)** and β-catenin **(D)** (*** *p* < 0.001 by unpaired t test with Welch’s correction). **(E-H)** The WB bands **(E)** and scaled normalized protein expressions of DSC2 **(F)**, ZO-1 **(G)** and OCLN **(H)** (** *p* < 0.01 by Mann Whitney test). **(I-L)** The relative mRNA expression of *Dsc2* **(I)**, *Cln3* **(J)**, *Tjp1* **(K)** and *Ocln* **(L)** (** *p* < 0.01, **** *p* < 0.0001 by unpaired t test with Welch’s correction). **(M, N)** Indexes of spleen **(M)** and thymus **(N)** (* *p* < 0.05, ** *p* < 0.01 by unpaired t test with Welch’s correction). The circles on the bar plots represent individual values with mean ± SEM (bars) (*n* = 5 rats/group).

Immunofluorescence staining showed that the duodenal expressions of E-cadherin and β-catenin were significantly decreased in the model group (**Figures 2A-D**). As shown in **Figures 2E-H**, the relative protein expressions of desmocollin 2 (DSC2), tight junction protein 1 (ZO-1) and occludin (OCLN) were markedly reduced in the model group. Moreover, significant decreases in the relative mRNA expressions of *Dsc2*, *Cln3*, *Tjp1* and *Ocln* were observed in the model group (**Figures 2I-L**). Concomitantly, indexes of spleen and thymus were decreased in the model group, indicating the compromise of immune function (**Figures 2M, N**).

### Duodenal microbiome regulated the biosynthesis of lipopolysaccharide and peptidoglycan in the rat model of FD

As for now, no available data on the characteristics of duodenal microbiome of FD. For reference, we performed a pooled analysis including two studies on the fecal microbiome of FD following the same modelling approach. After removing the batch effect, reanalysis of external datasets (BioProject ID: PRJNA575916, PRJNA719295) was conducted using the QIIME pipeline, which was the same for the subsequent analysis. The groups did not differ significantly in both alpha- and beta-diversity from each other. Nevertheless, function prediction of faecal microbiome suggests that the lipopolysaccharide biosynthesis was markedly elevated in the model group (**Supplemental Figure 1**).

**Figure 3:**
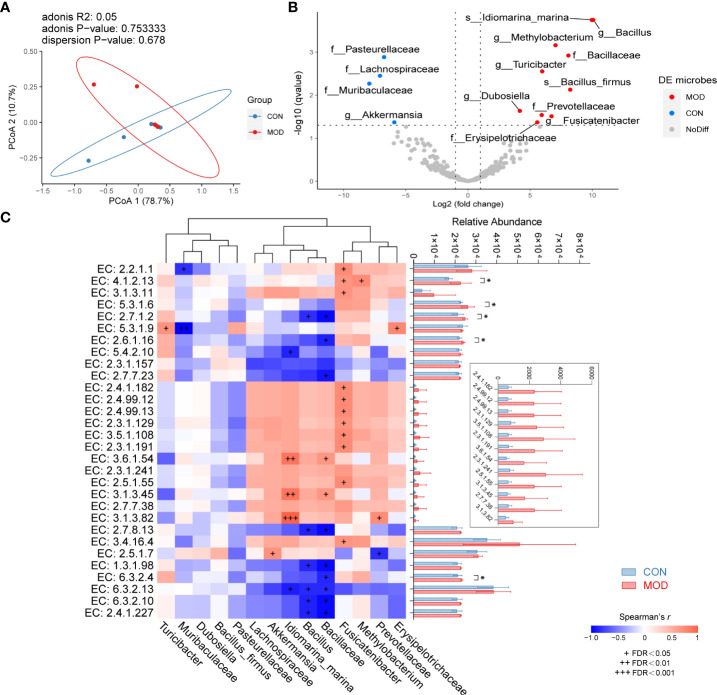
Microbial features of duodenum. **(A)** Weighted UniFrac PCoA with an adonis test. **(B)** Differential microbiome with DESeq2 analysis. The left dots (blue) indicate the microbiome enriched in control group; the right dots (red) are representative of the microbiome enriched in model group. **(C)** Spearman correlations of differential microbiome and predicted functional enzymes (Spearman’s *r* > 0.6 with + FDR < 0.05, ++ FDR < 0.01 or +++ FDR < 0.001 represent significant). The right side indicates multiple comparison of relative abundance of enzymes with Fisher’s LSD (mean ± SEM, * *p* < 0.05 represent significant) (*n* = 5 rats/group).

With focus on the duodenal microbial environment, we analysed the microbial composition and further predicted the metabolic enzymes of microbiome in our own experimental data. Similar to the previous results, no significant differences were found between the groups for both alpha- and beta-diversity (**Figure 3A**; **Supplemental Figure 2**). Differentially microbial species (the relative abundance of OTUs) were determined using DESeq2. Among these, *Pasteurellaceae*, *Lachnospiraceae*, *Muribaculaceae* identified to the family level and *Akkermansia* identified to the genus level were enriched in the control group, while *Bacillaceae*, *Prevotellaceae*, *Erysipelotrichaceae* identified to the family level, *Bacillus*, *Methylobacterium*, *Turicibacter*, *Dubosiella*, *Fusicatenibacter* identified to the genus level and *Idiomarina_marina*, *Bacillus_firmus* identified to the species level were enriched in the model group (**Figure 3B**). The detailed information of differential microbiome was summarized in **Supplemental Table 1**. Based on the PICRUSt2 analysis, we selected potential metabolic enzymes involved in the biosynthesis of lipopolysaccharide and peptidoglycan of duodenal microbiome (**Supplemental Figure 3**; **Supplemental Table 2**). Most of enzymes were elevated in the model group, although only a few of them reached statistical significance (**Figure 3C**). Spearman correlation coefficients between every two variables were calculated and were presented in **Figure 3C**; details were summarized in **Supplemental Table 3.**

### The upregulated innate immune response-related transcript profiles of the duodenum were associated with the proinflammatory toll-like receptor signaling pathway

**Figure 4:**
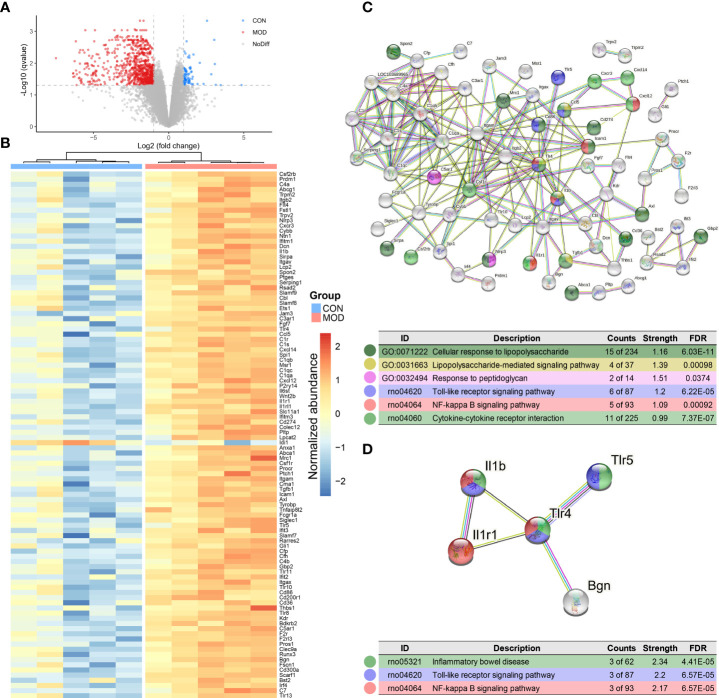
Transcriptome analysis of duodenum. **(A)** Differential expressed genes with DESeq2 analysis. Dotted line: BH FDR < 0.05 and |log2(FC)| > 1 represent significant (the control group: high expression in blue; the model group: high expression in red). **(B)** Selected differential genes based on InnateDB database. **(C)** PPI network based on innate immune response-related protein-coding genes (interaction score > 0.7 and hiding disconnected nodes). **(D)** The subnetwork clustering with MCL algorithm. Color-coded dots were clustered with GO and KEGG pathway enrichment analysis (*n* = 5 rats/group).

Given the potential DAMPs, we further explored the innate immune specific transcriptional signature of the duodenal mucosa. A total of 64 differentially expressed genes were upregulated in the control group while 835 further genes were significantly upregulated in the model group (**Figure 4A**). Based on these results, differentially expressed genes were filtered for innate immune responses using the InnateDB database. In total, 101 genes were significantly upregulated and only one was downregulated in the model group (**Figure 4B**). Details were presented in **Supplemental Table 4**. These protein-coding genes were matched with the STRING database for further interaction and enrichment analysis. PPI network was established and illustrated in **Figure 4C**. Then, subnetwork was established using MCL clustering (**Figure 4D**). Functional enrichment analysis indicated that the duodenal innate immune responses to lipopolysaccharide and peptidoglycan, which highlighted the importance of Toll-like receptor signaling and NK-κB-mediated proinflammatory effects.

### TLR2/TLR4-NFκB signaling and proinflammatory cytokines were elevated in the duodenum

**Figure 5:**
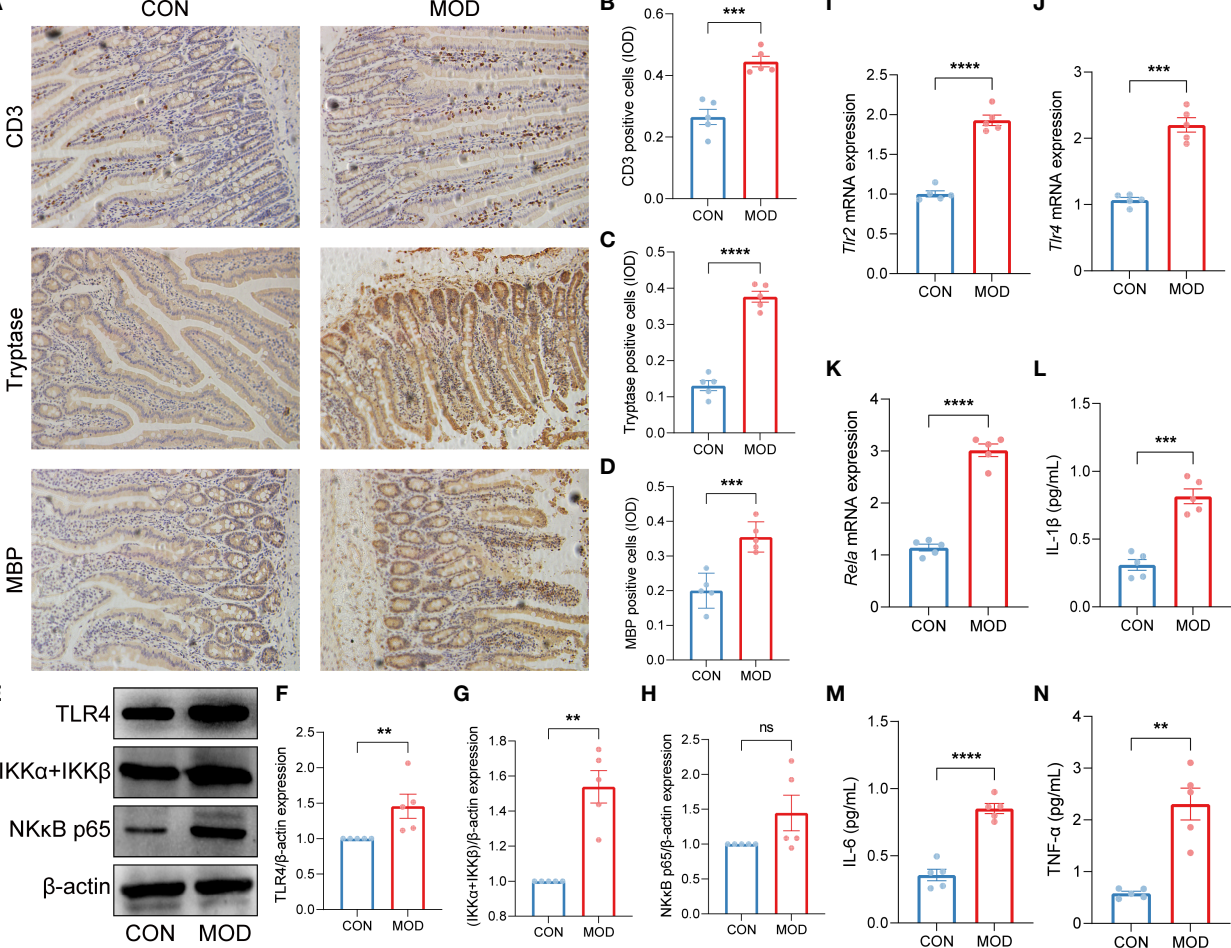
Validation of innate immune cells-mediated TLR2/TLR4-NFκB signaling pathway. **(A-D)** Duodenal immunohistochemical staining **(A)** and the relative expression (IOD) of CD3-labeled T lymphocytes **(B)**, tryptase-labeled mast cells **(C)** and MBP-labeled eosinophils **(D)** (*** *p* < 0.001, **** *p* < 0.0001 by unpaired t test with Welch’s correction). **(E-H)** The WB bands **(E)** and scaled normalized protein expressions of TLR4 **(F)**, IKKα + IKKβ **(G)** and NK-κB p65 **(H)** (** *p* < 0.01 by Mann Whitney test, ns indicates not significant). **(I-K)** The relative mRNA expressions of *Tlr2* **(I)**, *Tlr4* **(J)** and *Rela* **(K)** (*** *p* < 0.001, **** *p* < 0.0001 by unpaired t test with Welch’s correction). **(L-N)** The plasma contents of proinflammatory cytokines IL-1β **(L)**, IL-6 **(M)** and TNF-α **(N)** (IL-1β and IL-6: *** *p* < 0.001, **** *p* < 0.0001 by unpaired t test with Welch’s correction; TNF-α: ** *p* < 0.01 by Mann Whitney test) (*n* = 5 rats/group).

Immunohistochemical stain highlighted the increased CD3^+^ T lymphocyte, mast cell and eosinophil populations in the duodenum of the model group (**Figures 5A-D**). Western Blot analysis demonstrated significant elevations in the relative protein expressions of Toll-like receptor 4 (TLR4) and inhibitor of NFκB kinase subunits alpha and beta (IKKα+IKKβ) in the model group, but only NF-κB p65 subunit was significantly increased (**Figures 5E-H**). In addition, the relative mRNA expressions of *Tlr2*, *Tlr4* and *Rela* were markedly increased in the model group (**Figures 5I-K**). Subsequently, the levels of proinflammatory cytokines IL-1β, IL-6 and TNF-α were significant elevated in the model group (**Figures 5L-N**).

### The rat model of FD demonstrated a distinct metabolic pattern in the duodenum

**Figure 6:**
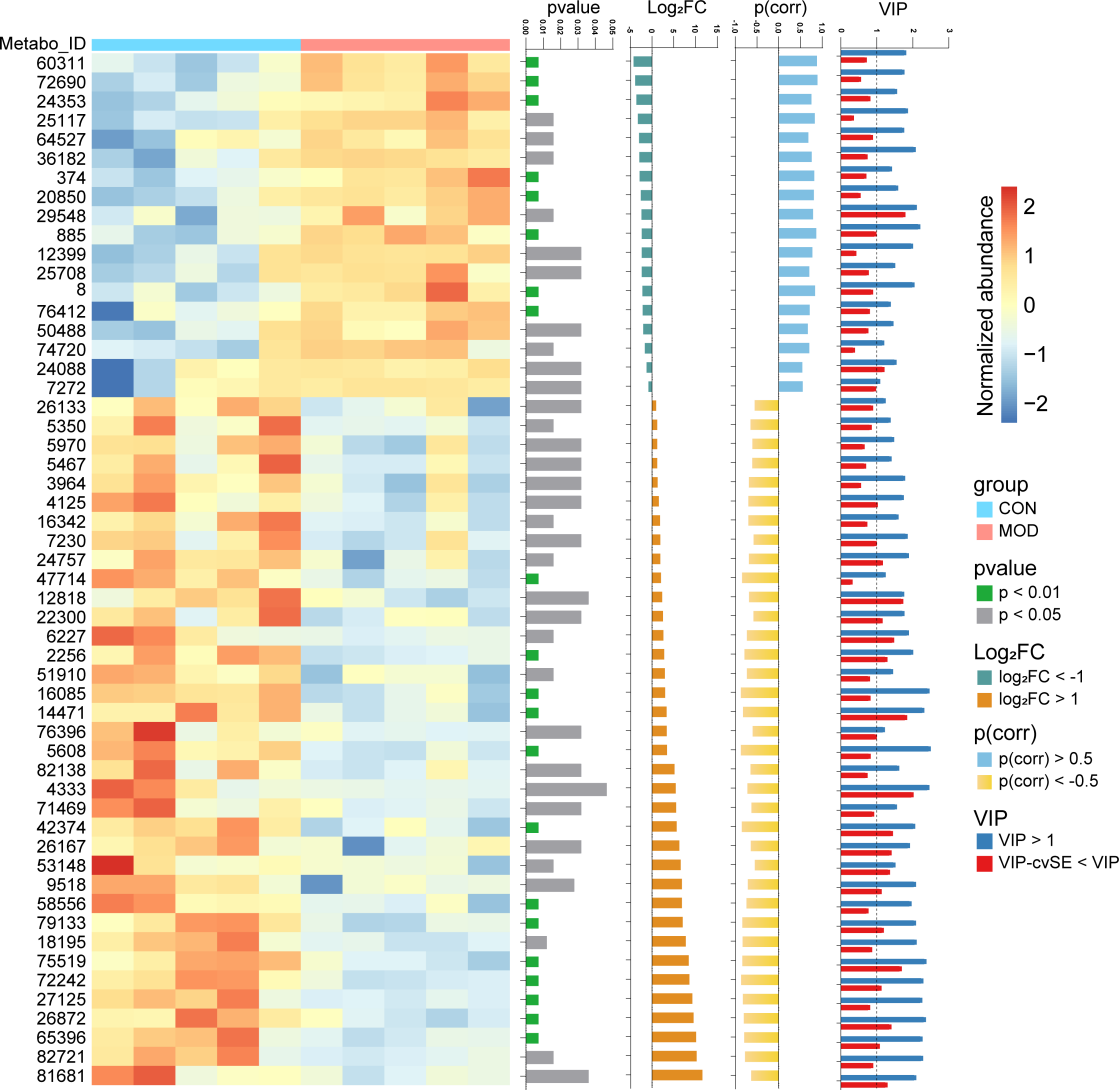
Metabolome analysis of duodenum. Differential metabolites were identified with thresholds of *p* < 0.05, |log2(FC)| > 1, |p(corr)| > 0.5 and VIP > 1 with cvSE of VIP less than the VIP value (*n* = 5 rats/group). The compound names corresponding to the Metabo_ID from top to bottom are as follows. 60311: Glycolithocholic acid; 72690: Taurolithocholate; 24353: Decanoyl-L-carnitine; 25117: Sphingosine; 64527: Glycodeoxycholic acid; 36182: Arachidonyl Ethanolamide; 374: Tyramine; 20850: C17 Sphingosine; 29548: Taurocholic acid; 885: Benzyl sulfate; 12399: Myristoyl Ethanolamide; 25708: Sphinganine; 8: 3-Hydroxybutyric acid; 76412: Taurodeoxycholate; 50488: Palmitoylcarnitine; 74720: Tricaprylin; 24088: Phytosphingosine; 7272: N,N-Dimethyltetradecylamine; 26133: 7-Sulfocholic acid; 5350: FA 18:2+O; 5970: Eicosapentaenoic acid; 5467: 9-HODE; 3964: gamma-Linolenic acid; 4125: Linoleic acid; 16342: Cholic acid; 7230: FA 18:2+2O; 24757: LPE(18:2/0:0); 47714: 7-Ketolithocholic acid; 12818: Lithocholic acid; 22300: Andrastin C; 6227: Arachidonic acid; 2256: Biotin; 51910: 6,7-Diketolithocholic acid; 16085: 3-Oxocholic acid; 14471: Murocholic acid; 76396: Taurochenodeoxycholate; 5608: FA 18:1+1O; 82138: LPC(18:1/0:0); 4333: Oleic acid; 71469: LPE(18:1/0:0); 42374: 3-Ketocholanic Acid; 26167: Oleoyl Ethanolamide; 53148: Hyocholic acid; 9518: N,N-Dimethyltetradecylamine-N-oxide; 58556: Stearoyl-L-Carnitine; 79133: LPC(17:0/0:0); 18195: Palmitoleoyl Ethanolamide; 75519: LPC(16:0/0:0); 72242: LPC(15:0/0:0); 27125: Docosahexaenoic acid; 26872: Stearoyl Ethanolamide; 65396: LPE(16:0/0:0); 82721: LPC(18:0/0:0); 81681: LPC(18:2/0:0).

In order to explore the factors that might mediate duodenal low-grade inflammation in the rat model of FD, we next performed untargeted metabolomics analysis of these duodenal tissues. Data were acquired in both positive and negative ion modes, respectively. In the positive ion mode, representative chromatograms of the control and model group were shown (**Supplemental Figures 4A, B**). After preprocessing the raw data, all samples with QC were analyzed using PCA (**Supplemental Figure 4C**). The OPLS-DA model was then established (**Supplemental Figure 4D**). Hotelling’s T^2^, Residuals Normal Probability and Permutation tests were used to evaluate the model (**Supplemental Figures 4E-G**). The VIP scores and correlation coefficients were acquired based on the OPLS-DA model (**Supplemental Figure 4H**). The same analysis for the negative ion mode was shown in **Supplemental Figure 5**. With additional thresholds, a total of 36 differential metabolites were enriched in the control group and 18 differential metabolites were enriched in the model group (**Figure 6**). The detailed information of metabolites was summarized in **Supplemental Table 5**.

### Arachidonyl ethanolamide and endocannabinoid analogues showed linear relationships with phenotypes found in FD

**Figure 7:**
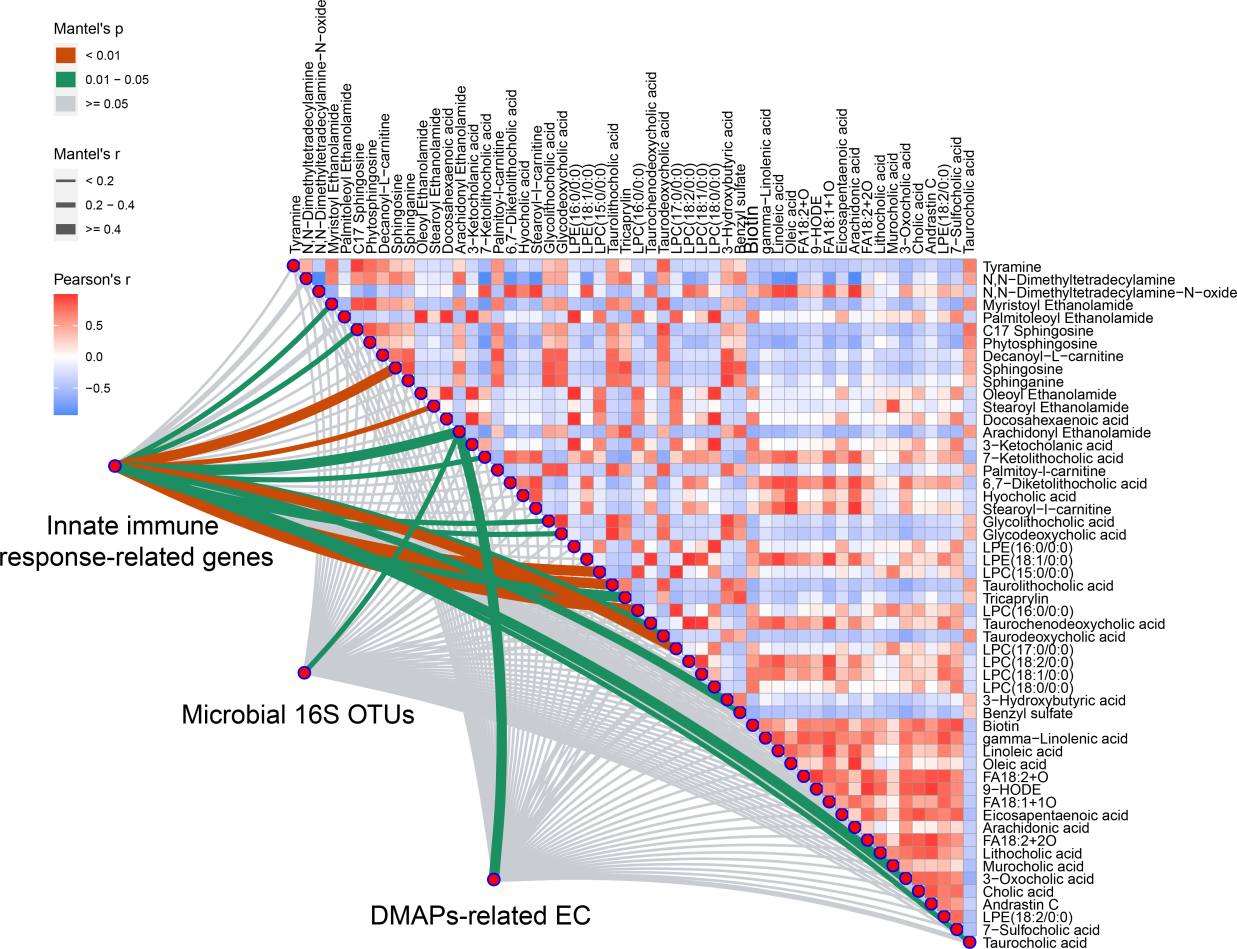
Variations of microbiome with DAMPs-related enzymes and duodenal metabolites have significant relationship with innate immune response-related genes expression. Integrated analysis of multi-omics data was performed using Mantel test. AEA was selected from all the three profiles (Mantel’s *p* < 0.05 represent significant) (*n* = 5 rats/group).

To integrate multi-level omics data, we conducted the mantel test when differential duodenal metabolites were used as environmental factors. As illustrated in **Figure 7**, AEA was the best explanatory variable for all the three profiles (the innate immune response-related genes: Mantel’s r = 0.503, p = 0.018; the microbial 16S OTUs: Mantel’s r = 0.310, p = 0.046; the DAMPs-related ECs: Mantel’s r = 0.468, p = 0.017). Details for calculation were summarized in **Supplemental Table 6**.

**Figure 8:**
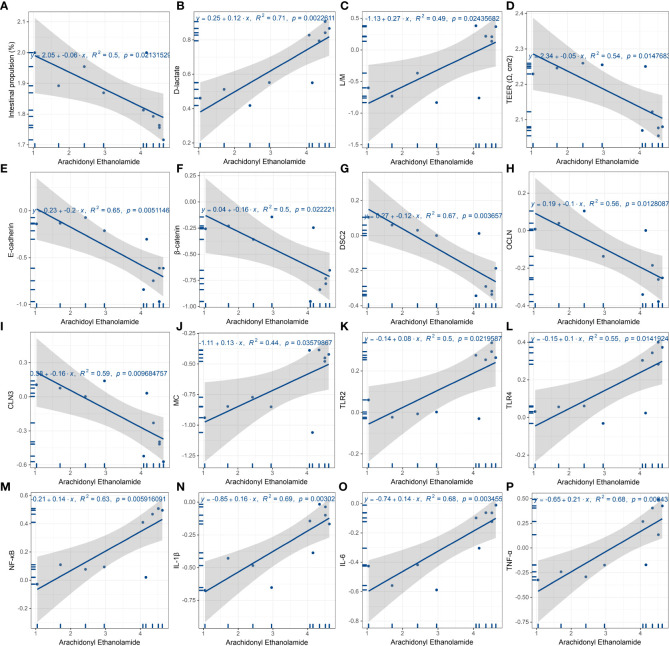
Linear regression analysis of AEA and features of FD. **(A)** Gastrointestinal motor function. AEA was negatively linear correlated with the gastrointestinal transit rate. **(B-D)** Duodenal permeability and absorption. AEA was positively linear correlated with L/M ratio **(B)** and the content of D-lactate **(C)**, while negatively linear correlated with TEER **(D)**. **(E-I)** Duodenal mucosal barrier. AEA was negatively linear corelated with the relative protein expressions of E-cadherin **(E)** and β-catenin **(F)** and the relative mRNA expressions of *Dsc2* **(G)**, *Ocln* **(H)** and *Cln3* **(I)**. **(J-P)** Innate immune cells and proinflammatory signaling. AEA was positively linear correlated with the relative amounts of mast cells **(J)**, the relative mRNA expressions of *Tlr2* **(K)**, *Tlr4* **(L)** and *Rela* **(M)**, and the contents of proinflammatory cytokines IL-1β **(N)**, IL-6 **(O)** and TNF-α **(P)** (All data are log10 transformed. *p* < 0.05 represent significant) (*n* = 5 rats/group).

Next, linear regression models were performed to present linear relationships between the relative abundance of AEA and phenotypes found in the rat model of FD. In gastrointestinal motor function, AEA was negatively linear correlated with the gastrointestinal transit rate (**Figure 8A**). With regards to permeability and absorption, AEA was positively linear correlated with L/M ratio and the content of D-lactate, and was negatively linear correlated with TEER (**Figures 8B-D**). In terms of duodenal mucosal barrier, AEA was negatively linear corelated with the relative protein expressions of E-cadherin and β-catenin and the relative mRNA expressions of DSC2, OCLN and CLN3 (**Figures 8E-I**). Additionally, AEA was positively linear correlated with the relative amounts of mast cells, the relative mRNA expressions of TLR2, TLR4 and NFκB, and the levels of proinflammatory cytokines IL-1β, IL-6 and TNF-α (**Figures 8J-P**). Aside from AEA, several endocannabinoid analogues, such as myristoyl ethanolamide (MEA), oleoyl ethanolamide (OEA), palmitoleoyl ethanolamide (PEA) and stearoyl ethanolamide (SEA), were also used to established linear regression models (**Supplemental Figures 6**-**9**). Instead, OEA, PEA and SEA were opposite to AEA described above.

## Discussion

Short-term gavage of iodoacetamide was firstly reported in 2008 by Liu and co-workers as the most classical modeling approach (9). Afterward, pharmacological and nonpharmacological therapies, including drug molecules, natural products and electroacupuncture, were extensively studied (10–12). The heterogeneity of FD has been demonstrated with different pathophysiological mechanisms under varied symptom conditions (13). Single-setting studies lack representativeness and comprehensiveness to understand the disease itself. Current review highlights the importance of the application of multi-omics methods such as the metabolomics data and the integration of these multiple layers in relation to phenotypes found in complex diseases (14). Unfortunately, no previous studies have compared and assessed the intertwined characteristics using a multi-omics approach.

A recent study with a large sample size reported FD and other gastrointestinal disorders shares several commonalities in a wide spectrum of pathophysiology, which increases the difficulty of treatment against a specific disease (15). Compared to a global exploration, it is more important to profile the comprehensive features locally. One study has demonstrated that TEER and the expression of ZO-1 are significantly decreased in patients with abdominal symptoms of FD. And the level of IL-1β elevated in the patients is negatively correlated with both of above measures (16). Another clinical study using confocal laser endomicroscopy visually confirmed that the impairment of duodenal mucosal barrier was an important pathogenesis factor in FD (4). Similar to these results, we also found that TEER was significantly decreased and the same trend as the relative mRNA expressions of tight junction proteins in the rat model of FD. Moreover, an elevated trend of the levels of proinflammatory cytokines IL-1β, IL-6 and TNF-α in the clinical samples were in line with our *in vivo* study data. The activation of T lymphocyte and eosinophilia with increased peripheral proinflammatory cytokines IL-1β, IL-6 and TNF-α are identified as the main features of FD (17). The duodenal hyperplasia of mast cells and eosinophils have been reported as the pathophysiological phenomena overlapping irritable bowel syndrome and FD as demonstrated in a cross-sectional study (18). As an administration of corticotropin-release hormone, the mast cell-eosinophil signalling increases small intestinal permeability (19). In addition, mast cells and eosinophilia in activation statue have been observed to cluster around intestinal submucosal plexus neurons, which alter the neuronal responsiveness of intestine and delayed gastric emptying (20, 21).

In terms of the initial triggers involved in the pathophysiological process, one view emphasizes the eating-related symptoms and the dietetic management of FD (22). The duodenal microbes support the digestive functions of small intestine with the actions of fermentation and non-host functional enzymes, which prevent inappropriate activation of immune responses towards foods (23). Beneficial immune and microbial regulation and the treatment of FD with specific probiotics have also been demonstrated to be effective and safe (24). The colonized microbes serve as a signalling hub that incorporate environmental exposure and signals, regulating the host’s metabolism and innate immune system (25). On the other hand, host innate immunity regulates the microbial distribution along the gastrointestinal tract. Importantly, the microbiome located in duodenal mucosa indicate a greater sensitivity to the innate immune responses compared to other intestinal sites (26). The duodenal microenvironment has emerged as an important player in the pathophysiological mechanisms of FD, in which both locally microbial community disorder, host and microbial metabolism and host innate immunity are involved (27). These imbalances may in part be mediated by specific microbiome-associated metabolites (28). A recent study showed that the metabolic functional prediction of oral and gastric microbiome based on 16S rRNA sequencing data. Among these, purine metabolism, biosynthesis of lipopolysaccharide and amino acid related enzymes were enriched in saliva microbiome, while peptidases and associated processing at the gastric level. However, metabolic function of duodenal microbiome has not been reported (29).

Previous clinical data showed that increased fasting plasma AEA has a significant negative correlation with the duodenal expression of ZO-1. There was a similar, although non-significant, trend in the relationship between AEA and the relative mRNA expression of TLR4 and the content of TNF-α, while an opposite trended association between AEA and ZO-1 (30). Another clinical study using positron emission tomography have demonstrated the higher availability of endocannabinoid 1 (CB1) receptor in the different cerebral regions of patients with FD, indicating that the dysfunction of endocannabinoid system is involved in the disease process (31). Additionally, enteroendocrine cells in duodenum also contain the main mRNA transcripts that encode endocannabinoids and biosynthesis-associated fatty acids (32). In healthy populations, pre-treatment with the CB1 receptor antagonist inhibited the gastric accommodation reflex but not compliance, distension and nutrient tolerance (33). The application of endocannabinoid receptor antagonist *in vitro* significantly decreased TEER and the relative mRNA expressions of ZO-1 and OCLN (34). Animal study has also proved that addition of endocannabinoid receptor antagonist elevates the intestinal permeability (35). Some notions have been substantiated that AEA can be synthesized from mast cells (36) and lymphocytes (37). Furthermore, CB1 receptor is expressed on the surface of mast cells (38), while both CB1 and CB2 receptors on eosinophils (39, 40) and T lymphocytes (41, 42). As the gate opener assisting actions of DAMPs, AEA exerts negative effects on the intestinal barrier, presenting the gut microbiota-endocannabinoid system axis in relation to the host metabolism (5). In contrast, several endocannabinoid analogues have been recognised as the key gate keepers. With the region-specific actions, intestinal OEA plays a major role in gut physiological processes in the host (43). As the natural agonist of peroxisome proliferator-activated receptor-α (PPARα) with a high affinity, OEA effectively relieves peripheral inflammatory conditions (44). Similarly, the PEA of leukocytes actives PPARα at the early stage of LPS-induced inflammation in a PPARα-dependent manner (45, 46). The reduced levels of OEA, PEA and SEA also disturbed lipid and fatty acid metabolism, resulting in proinflammatory responses (47). Taken together, the specific alternation of intestinal immunity has associations with endocannabinoid system, microbial community and host metabolism (48). Nevertheless, research study on the intertwined correlation among the above three layers has not been reported.

Our study has some shortcomings. Due to technical limitations, clarified protein-coding genes of duodenal microbiome could not be confirmed. Verification studies on host endocannabinoid system are needed, which are currently on-going. Further studies at the cellular level, and clinical data are also needed to suggest how the endocannabinoid system regulates the cause-effect relationship between microbial metabolic function and host innate immune response.

In conclusion, this study provides multi-omics evidence to suggest that duodenal microbiome regulate the biosynthesis of lipopolysaccharide and peptidoglycan; and the host endocannabinoid system acts as the potential regulator of duodenal DAMPs-mediated low-grade inflammation in the rat model of FD.

## Methods

### Animal model

The rat model of FD was developed as described previously (9). In brief, acclimatization lasted one week after the quarantine period of 3 days. In the model group, the oral gavage with 200μl of 0.1% iodoacetamide solution (dissolved in 2% sucrose aqueous solution) was administered once daily for 6 days, while the control group was given normal 2% sucrose aqueous solution at the same time. All the rats were fed on a standard chow up to 8 weeks of age. The duodenal tissues of additional two rats from each group were examined using transmission electron microscopy. Another three rats from each group were used for the assessment of behavioural response to gastric distention due to any potential confounders related to proinflammatory responses.

### Behavioral response to gastric balloon distention

The balloon fabrication procedure, implantation surgery and behavioral response to GD were performed according to previous study (9). After 24-hour fasting, 8-week-old rats of model group were anaesthetized with 10% chloral hydrate intraperitoneally. Balloons with surface sterilization should not obstruct the pylorus. Behavioral testing of GD was performed on day 6 after surgery. The rats of model group were allowed an hour to acclimate to the individual plastic environment. The inflating pressure was slowly increased to 20, 40, 60, 80 mmHg for 20 seconds with 5 minutes rest, respectively. The behavioral response to GD was graded into 0–4 rating scale as described previously.

### Gastrointestinal transit

The calculation of gastrointestinal transit referenced the previous study (49). Briefly, all the rats were gavaged with a charcoal marker (mixtures of 20% charcoal and 5% gum arabic, 1mL/100g body weight) into the stomach after 24-hour fasting. Following the formula, the gastrointestinal transit rate (%) = migration distance of charcoal marker/whole length of the small intestine × 100%. Each measurement was repeated three times and the mean value was taken.

### Trans-endothelial electrical resistance

A Ussing chamber was used to measure the TEER as described previously (50) with some modifications. Fresh duodenum tissues (1cm long × 0.5cm wide) were cleaned and rapidly put into Krebs-Ringer buffer with constant carbogenation (O_2_/CO_2_, 95/5%) at 37°C. The parameters were set as stable voltage of 5mV and polar constant-current pulses of 16 mA every 60 s with a 200 ms duration. Measurements were recorded every 30 min within 2 h.

### Quantitation of lactulose and mannitol

Duodenal permeability was assessed by L/M ratio using high performance liquid chromatography (HPLC) Agilent 1260 (Agilent Technologies, Santa Clara, CA, USA) with a Waters C18 column (Waters, Milford, USA), referring to previous literature (51) with adjustment according to our laboratory experience.

### Immune organ index

The innate immune-related organs (thymus and spleen) were collected and freshly weighed. Excess liquid was blotted off using filter paper to reduce computational error. The immune organ index was calculated by the following formula: organ index (%) = organ weight/body weight × 100%.

### Quantitation of D-lactate and cytokines

Plasma D-lactate and proinflammatory cytokines were quantized using enzyme-linked immunosorbent assays (ELISA). EnzyChrom™ D-Lactate Assay Kit was purchased from BioAssay Systems (Hayward, CA). Rat IL-1 beta ELISA Kit (ab255730), IL-6 ELISA Kit (ab234570) and TNF alpha ELISA Kit (ab236712) were purchased from Abcam (Shanghai, China). Establishment of standard curves and specific experimental steps followed the instructions of above ELISA kits. The optical density (OD) values of D-Lactate and cytokines (IL-1β, IL-6 and TNF-α) were respectively read at 565 and 450 nm.

### Immunohistochemical and immunofluorescence staining

*IHC*. A portion of the duodenum tissue was processed for paraffin embedding. Immunohistochemical staining for CD3 (52), mast cell tryptase (53) and eosinophil MBP (54) were performed as described previously. Anti-CD3 (ab5690) and anti-mast cell tryptase (ab2378) antibodies were purchased from Abcam (Shanghai, China). Anti-eosinophil major basic protein antibody (MBP) [BMK-13] (ARG22591) was purchased from arigo Biolaboratories (Shanghai, China).

*IF*. Immunofluorescent labeling of duodenal sections with antibodies against β-Catenin (55) and E-cadherin (56) was performed as previous studies. β-Catenin (D10A8) XP^®^ Rabbit mAb (#8480), E-Cadherin (4A2) Mouse mAb (#14472), anti-mouse IgG (H+L), F(ab’)2 Fragment (Alexa Fluor^®^ 488 Conjugate) (#4408), anti-rabbit IgG (H+L), F(ab’)2 Fragment (Alexa Fluor^®^ 555 Conjugate) (#4413) were purchased from Cell Signaling Technologies (Shanghai, China).

For each specimen, three random visual fields as well as three duplications were observed. The integrated optical density (IOD) was calculated using Image-Pro Plus 6.0 software.

### Transmission electron microscopy analysis

For intuitively observing tight junctions between epithelial cells, additional fresh duodenum tissues (chopped in 1mm^3^ pieces) of each group were fixed with pre-cooled 2.5% glutaraldehyde. Standard procedures of TEM and image processing referred to previous study (57).

### 16S rRNA sequencing

After 24-hour fasting, duodenum tissues with contents were aseptically retrieved. Considering microbiome located in the crypt-villus structure, duodenum tissues and contents were mixed with the homogenate. The sodium dodecyl sulfate (SDS) with grinding-based method was used to extract genomic DNA. After purifying with GeneJET Gel Extraction Kit (Thermofisher), PCR product was collected to generate libraries of the V4 region (PCR primers: 515F/806R (58)) using Ion Plus Fragment Library Kit 48 rxns (Thermofisher). Sequencing was performed on the Ion S5™XL platform (Thermofisher). Raw SE400 reads were filtered with Cutadapt (V1.9.1, http://cutadapt.readthedocs.io/en/stable/). Chimeras were identified and removed with vsearch (V1.7.0, https://github.com/torognes/vsearch). Clean reads obtained as described above were clustered using Uparse (Uparse v7.0.1001, http://drive5.com/uparse/) to obtain operational taxonomic units (OTUs) with a threshold of 97% similarity. Species annotation was performed on the representative sequences of OTUs using Mothur (https://mothur.org/), on which SSUrRNA database of SILVA (Release 132) (http://www.arb-silva.de/) was used with a threshold of 0.8~1). Multiple sequence alignment was conducted to investigate phylogenetic relationship by MUSCLE (v3.8.31, http://www.drive5.com/muscle/). Alpha and beta diversity metrics were analyzed using QIIME (V1.9.1) pipeline. Functional enzymes and KEGG pathways prediction was analyzed using PICRUSt2 (https://github.com/picrust/picrust2). The publicly available datasets can be download through NCBI SRA database (BioProject ID: PRJNA575916, PRJNA719295). In-house raw data are also available for download (BioProject ID: PRJNA835600).

### Transcriptome sequencing

Total RNA was extracted with Trizol (Invitrogen). The magnetic beads with Oligo (dT) were used to enrich mRNA. Concentration and purity were determined based on 260/280nm UV absorbance ratios and the integrity was then measured by Agilent 2100 bioanalyzer. The transcriptome libraries were obtained using NEBNext^®^ Ultra™ RNA Library Prep Kit for Illumina^®^ (NEB). Sequencing was performed on the Hiseq 4000 (Illumina) platform. After filtering, clean reads were aligned to the reference genome using HISAT2 (https://daehwankimlab.github.io/hisat2/). Transcripts were then assembled with StringTie (http://ccb.jhu.edu/software/stringtie/). The resulting read counts were converted to FPKM value. The genes in relation to innate immunity were selected based on the InnateDB database (https://www.innatedb.ca/). The PPI network was established with STRING (http://string-db.org) with a minimum required interaction score > 0.7. A subnetwork was then clustered with a MCL algorithm. In-house raw data are available for download through NCBI SRA database (BioProject ID: PRJNA835595).

### Untargeted metabolome

Sample preprocessing steps and TOF MS paraments were the same as our previous described (59). The UPLC gradient was programmed with some modifications. Raw data was converted to abf using abfconvert. Based on MS-DIAL (V4.24) software, peak detection, deconvolution, samples alignment, compounds identification and computation of missing values were performed using an in-house method.

### Western blot

A BCA assay was used to measure the concentration of total protein extracted from homogenized duodenum tissues. Western blot was performed following standard procedures. Briefly, 20μl denatured protein each well was loaded onto SDS-PAGE. After electrophoresis, the proteins were electroblotted to a PVDF membrane. Incubations of the primary and secondary antibodies were performed overnight at 4°C and for one hour at room temperature, respectively. Anti-Tight Junction Protein 1 (ZO-1) antibody (NBP1-85047) was purchased from Novus (Shanghai, China). Anti-Occludin (OCLN) (ab167161), anti-Claudin 3 (CLN3) (ab15102), anti-Desmocollin 2 (DSC2) (ab230039), anti-Toll-like receptor 4 (TLR4) (ab217274), anti-IKK alpha + IKK beta (ab178870), anti-NK-κB p65 (ab16502) and anti-beta Actin (ab8227) antibodies were purchased from Abcam (Shanghai, China). After washing with TBST three times for 10 min, ECL detection, exposure and development were performed. Protein band intensity was measured and quantified by Image Lab software.

### Quantitative PCR

Purified mRNA described above was reverse transcribed to first-strand cDNA using an Evo M-MLV One Step RT-qPCR Kit (Accurate Biotechnology). Templates were collected following the condition of 37°C for 15 min and 85°C for 5 s. A SYBR^®^ Green Premix Pro Taq HS qPCR Kit (Accurate Biotechnology) was used to amplification following the condition of one cycle for 30 s at 95°C and 40 cycles for 5 s at 95°C, 30 s at 60°C. PCR product was quantified using comparative ΔCt method (relative quantification, RQ). Primers information was summarized in **Supplemental Table 7**.

### Statistics

Welch-corrected t test and Mann-Whitney test were conducted using GraphPad Prism (9.2.0). In beta diversity, adonis statistics were performed based on QIIME pipeline. With thresholds of BH FDR < 0.05 and |log2(FC)| > 1, differential expressed microbiome and genes were identified by DESeq2 (https://git.bioconductor.org/packages/DESeq2). For metabolomics data, multivariate analysis was performed using MetaboAnalyst (v5.0) and SIMCA (V14.1). Compounds with multiple thresholds of *p* < 0.05, |log2(FC)| > 1, |p(corr)| > 0.5 and VIP > 1 with cross-validation standard error (cvSE) of VIP less than the VIP value were selected as differential metabolites. Mantel test was performed to established connections with multi-omics data with Pearson correlation coefficient. The Spearman correlation coefficient was used to analyze the correlations between microbiome and functional enzymes with a threshold of *p* < 0.05.

### Study approval

All animal experiments were followed the standards of the Guide for the Care and Use of Laboratory Animals (National Research Council. 2011. Guide for the care and use of laboratory animals, 8th ed. National Academies Press, Washington, DC) and were approved by the Institutional Animal Care and Use Committee of Guangdong Provincial Hospital of Chinese Medicine (Ethics Approval Number: 2017007-2).

## Data availability statement

The datasets presented in this study can be found in online repositories. The names of the repository/repositories and accession number(s) can be found in the article/**Supplementary Material**.

## Ethics statement

The animal study was reviewed and approved by the Institutional Animal Care and Use Committee of Guangdong Provincial Hospital of Chinese Medicine.

## Author contributions

Acquiring and analyzing data were contributed by SJ and YY. Conducting experiments was contributed by BP, TZ, YK, LD and LC. Designing research was contributed by YY, SH, YW and YL. Funding acquisition was contributed by YL and JD. Investigation was contributed by YW, SL, SH and YW. Providing reagents was contributed by SL, SH and YW. Supervision was contributed by XS, JD, SH and YL. Writing the manuscript was contributed by SJ and BP. All authors reviewed and edited the manuscript.

## Funding

This work was supported by the Key Project of National Natural Science Foundation of China (81830117), the National Natural Science Foundation of China (81873205 and 81904037), the Natural Science Foundation of Guangdong Province, China (2019A1515010400, 2021A1515110990), the Science and Technical Plan of Guangzhou, Guangdong, China (201903010069), and the Innovation Team and Talents Cultivation Program of the National Administration of Traditional Chinese Medicine (ZYYCXTD-C-202001).

## Acknowledgments

We also thank Professor Hiu Yee Kwan for the language revision.

## Conflict of interest

The authors declare that the research was conducted in the absence of any commercial or financial relationships that could be construed as a potential conflict of interest.

## Publisher’s note

All claims expressed in this article are solely those of the authors and do not necessarily represent those of their affiliated organizations, or those of the publisher, the editors and the reviewers. Any product that may be evaluated in this article, or claim that may be made by its manufacturer, is not guaranteed or endorsed by the publisher.

## References

[1] Ford AC Mahadeva S Carbone MF Lacy BE Talley NJ. Functional dyspepsia. Lancet (2020) 396(10263):1689–702. doi: 10.1016/S0140-6736(20)30469-4 33049222

[2] Black CJ Drossman DA Talley NJ Ruddy J Ford AC. Functional gastrointestinal disorders: advances in understanding and management. Lancet (2020) 396(10263):1664–74. doi: 10.1016/S0140-6736(20)32115-2 33049221

[3] Cordner ZA Li Q Liu L Tamashiro KL Bhargava A Moran TH. Vagal gut-brain signaling mediates amygdaloid plasticity, affect, and pain in a functional dyspepsia model. JCI Insight (2021) 6(6):e144046. doi: 10.1172/jci.insight.144046 PMC8026195 33591956

[4] Nojkov B Zhou S-Y Dolan RD Davis EM Appelman HD Guo X. Evidence of duodenal epithelial barrier impairment and increased pyroptosis in patients with functional dyspepsia on confocal laser endomicroscopy and "Ex vivo" mucosa analysis. Am J Gastroenterol (2020) 115(11):1891–901. doi: 10.14309/ajg.0000000000000827 PMC8409129 33156108

[5] Cani PD Plovier H Van Hul M Geurts L Delzenne NM Druart C. Endocannabinoids–at the crossroads between the gut microbiota and host metabolism. Nat Rev Endocrinol (2016) 12(3):133–43. doi: 10.1038/nrendo.2015.211 26678807

[6] Di Marzo V Ligresti A Cristino L. The endocannabinoid system as a link between homoeostatic and hedonic pathways involved in energy balance regulation. Int J Obes (Lond). (2009) 33 Suppl 2:S18–24. doi: 10.1038/ijo.2009.67 19528974

[7] Di Marzo V Capasso R Matias I Aviello G Petrosino S Borrelli F. The role of endocannabinoids in the regulation of gastric emptying: alterations in mice fed a high-fat diet. Br J Pharmacol (2008) 153(6):1272–80. doi: 10.1038/sj.bjp.0707682 PMC2275439 18223666

[8] Izzo AA Piscitelli F Capasso R Aviello G Romano B Borrelli F. Peripheral endocannabinoid dysregulation in obesity: relation to intestinal motility and energy processing induced by food deprivation and re-feeding. Br J Pharmacol (2009) 158(2):451–61. doi: 10.1111/j.1476-5381.2009.00183.x PMC2757684 19371345

[9] Liu L-S Winston JH Shenoy MM Song G-Q Chen JDZ Pasricha PJ. A rat model of chronic gastric sensorimotor dysfunction resulting from transient neonatal gastric irritation. Gastroenterology (2008) 134(7):2070–9. doi: 10.1053/j.gastro.2008.02.093 18448102

[10] Liu LS Shenoy M Pasricha PJ. The analgesic effects of the GABAB receptor agonist, baclofen, in a rodent model of functional dyspepsia. Neurogastroenterol Motil. (2011) 23(4):35661.e160-1. doi: 10.1111/j.1365-2982.2010.01649.x 21199535

[11] Zhao J Zhao L Zhang S Zhu C. Modified liu-Jun-Zi decoction alleviates visceral hypersensitivity in functional dyspepsia by regulating EC cell-5HT3r signaling in duodenum. J Ethnopharmacol (2020) 250:112468. doi: 10.1016/j.jep.2019.112468 31836517

[12] Ouyang X Li S Zhou J Chen JD. Electroacupuncture ameliorates gastric hypersensitivity *via* adrenergic pathway in a rat model of functional dyspepsia. Neuromodulation (2020) 23(8):1137–43. doi: 10.1111/ner.13154 32282996

[13] Vanheel H Farré R. Changes in gastrointestinal tract function and structure in functional dyspepsia. Nat Rev Gastroenterol Hepatol (2013) 10(3):142–9. doi: 10.1038/nrgastro.2012.255 23318268

[14] Wörheide MA Krumsiek J Kastenmüller G Arnold M. Multi-omics integration in biomedical research - a metabolomics-centric review. Anal Chim Acta (2021) 1141:144–62. doi: 10.1016/j.aca.2020.10.038 PMC7701361 33248648

[15] Garcia-Etxebarria K Carbone F Teder-Laving M Pandit A Holvoet L Thijs V. A survey of functional dyspepsia in 361,360 individuals: Phenotypic and genetic cross-disease analyses. Neurogastroenterol Motil. (2021) 34(6):e14236. doi: 10.1111/nmo.14236 34378841

[16] Komori K Ihara E Minoda Y Ogino H Sasaki T Fujiwara M. The altered mucosal barrier function in the duodenum plays a role in the pathogenesis of functional dyspepsia. Dig Dis Sci (2019) 64(11):3228–39. doi: 10.1007/s10620-019-5470-8 30673985

[17] Burns G Carroll G Mathe A Horvat J Foster P Walker MM. Evidence for local and systemic immune activation in functional dyspepsia and the irritable bowel syndrome: A systematic review. Am J Gastroenterol (2019) 114(3):429–36. doi: 10.1038/s41395-018-0377-0 30839392

[18] Walker MM Talley NJ Prabhakar M Pennaneac'h CJ Aro P Ronkainen J. Duodenal mastocytosis, eosinophilia and intraepithelial lymphocytosis as possible disease markers in the irritable bowel syndrome and functional dyspepsia. Aliment Pharmacol Ther (2009) 29(7):765–73. doi: 10.1111/j.1365-2036.2009.03937.x PMC4070654 19183150

[19] Wallon C Yang PC Keita AV Ericson AC McKay DM Sherman PM. Corticotropin-releasing hormone (CRH) regulates macromolecular permeability *via* mast cells in normal human colonic biopsies in vitro. Gut (2008) 57(1):50–8. doi: 10.1136/gut.2006.117549 17525093

[20] Cirillo C Bessissow T Desmet A-S Vanheel H Tack J Vanden Berghe P. Evidence for neuronal and structural changes in submucous ganglia of patients with functional dyspepsia. Am J Gastroenterol (2015) 110(8):1205–15. doi: 10.1038/ajg.2015.158 26077177

[21] Liebregts T Adam B Bredack C Gururatsakul M Pilkington KR Brierley SM. Small bowel homing T cells are associated with symptoms and delayed gastric emptying in functional dyspepsia. Am J Gastroenterol (2011) 106(6):1089–98. doi: 10.1038/ajg.2010.512 21245834

[22] Duncanson K Burns G Pryor J Keely S Talley NJ. Mechanisms of food-induced symptom induction and dietary management in functional dyspepsia. Nutrients (2021) 13(4):1109. doi: 10.3390/nu13041109 33800668 PMC8066021

[23] Oliphant K Allen-Vercoe E. Macronutrient metabolism by the human gut microbiome: major fermentation by-products and their impact on host health. Microbiome (2019) 7(1):91. doi: 10.1186/s40168-019-0704-8 31196177 PMC6567490

[24] Wauters L Slaets H De Paepe K Ceulemans M Wetzels S Geboers K. Efficacy and safety of spore-forming probiotics in the treatment of functional dyspepsia: a pilot randomised, double-blind, placebo-controlled trial. Lancet Gastroenterol Hepatol (2021) 6(10):784–92. doi: 10.1016/S2468-1253(21)00226-0 34358486

[25] Thaiss CA Zmora N Levy M Elinav E. The microbiome and innate immunity. Nature (2016) 535(7610):65–74. doi: 10.1038/nature18847 27383981

[26] Gu M Samuelson DR de la Rua NM Charles TP Taylor CM Luo M. Host innate and adaptive immunity shapes the gut microbiota biogeography. Microbiol Immunol (2022) 66(6):330–41. doi: 10.1111/1348-0421.12963. PMC9189012 35067963

[27] Wauters L Talley NJ Walker MM Tack J Vanuytsel T. Novel concepts in the pathophysiology and treatment of functional dyspepsia. Gut (2020) 69(3):591–600. doi: 10.1136/gutjnl-2019-318536 31784469

[28] Burns GL Hoedt EC Walker MM Talley NJ Keely S. Physiological mechanisms of unexplained (functional) gastrointestinal disorders. J Physiol (2021) 599(23):5141–61. doi: 10.1113/JP281620 34705270

[29] Cervantes J Michael M Hong B-Y Springer A Guo H Mendoza B. Investigation of oral, gastric, and duodenal microbiota in patients with upper gastrointestinal symptoms. J Investig Med (2020) jim-2020-001642. doi: 10.1136/jim-2020-001642. 33335025

[30] Little TJ Cvijanovic N DiPatrizio NV Argueta DA Rayner CK Feinle-Bisset C. Plasma endocannabinoid levels in lean, overweight, and obese humans: relationships to intestinal permeability markers, inflammation, and incretin secretion. Am J Physiol Endocrinol Metab (2018) 315(4):E489–95. doi: 10.1152/ajpendo.00355.2017 PMC6230711 29438631

[31] Ly HG Ceccarini J Weltens N Bormans G Van Laere K Tack J. Increased cerebral cannabinoid-1 receptor availability is a stable feature of functional dyspepsia: a [F]MK-9470 PET study. Psychother Psychosom. (2015) 84(3):149–58. doi: 10.1159/000375454 25833408

[32] Sykaras AG Demenis C Case RM McLaughlin JT Smith CP. Duodenal enteroendocrine I-cells contain mRNA transcripts encoding key endocannabinoid and fatty acid receptors. PloS One (2012) 7(8):e42373. doi: 10.1371/journal.pone.0042373 22876318 PMC3410929

[33] Ameloot K Janssen P Scarpellini E Vos R Boesmans W Depoortere I. Endocannabinoid control of gastric sensorimotor function in man. Aliment Pharmacol Ther (2010) 31(10):1123–31. doi: 10.1111/j.1365-2036.2010.04259.x 20146701

[34] Muccioli GG Naslain D Bäckhed F Reigstad CS Lambert DM Delzenne NM. The endocannabinoid system links gut microbiota to adipogenesis. Mol Syst Biol (2010) 6:392. doi: 10.1038/msb.2010.46 20664638 PMC2925525

[35] Matias I Gonthier M-P Orlando P Martiadis V De Petrocellis L Cervino C. Regulation, function, and dysregulation of endocannabinoids in models of adipose and beta-pancreatic cells and in obesity and hyperglycemia. J Clin Endocrinol Metab (2006) 91(8):3171–80. doi: 10.1210/jc.2005-2679 16684820

[36] Bisogno T Maurelli S Melck D De Petrocellis L Di Marzo V. Biosynthesis, uptake, and degradation of anandamide and palmitoylethanolamide in leukocytes. J Biol Chem (1997) 272(6):3315–23. doi: 10.1074/jbc.272.6.3315 9013571

[37] Maccarrone M De Petrocellis L Bari M Fezza F Salvati S Di Marzo V. Lipopolysaccharide downregulates fatty acid amide hydrolase expression and increases anandamide levels in human peripheral lymphocytes. Arch Biochem Biophys (2001) 393(2):321–8. doi: 10.1006/abbi.2001.2500 11556820

[38] Sugawara K Zákány N Hundt T Emelianov V Tsuruta D Schäfer C. Cannabinoid receptor 1 controls human mucosal-type mast cell degranulation and maturation in situ. J Allergy Clin Immunol (2013) 132(1):182–93. doi: 10.1016/j.jaci.2013.01.002 23453134

[39] Small-Howard AL Shimoda LMN Adra CN Turner H. Anti-inflammatory potential of CB1-mediated cAMP elevation in mast cells. Biochem J (2005) 388(Pt 2):465–73. doi: 10.1042/BJ20041682 PMC1138953 15669919

[40] Chouinard F Lefebvre JS Navarro P Bouchard L Ferland C Lalancette-Hébert M. The endocannabinoid 2-arachidonoyl-glycerol activates human neutrophils: critical role of its hydrolysis and *de novo* leukotriene B4 biosynthesis. J Immunol (2011) 186(5):3188–96. doi: 10.4049/jimmunol.1002853 21278347

[41] Castaneda JT Harui A Kiertscher SM Roth JD Roth MD. Differential expression of intracellular and extracellular CB(2) cannabinoid receptor protein by human peripheral blood leukocytes. J Neuroimmune Pharmacol (2013) 8(1):323–32. doi: 10.1007/s11481-012-9430-8 PMC3587044 23299999

[42] Sánchez López AJ Román-Vega L Ramil Tojeiro E Giuffrida A García-Merino A. Regulation of cannabinoid receptor gene expression and endocannabinoid levels in lymphocyte subsets by interferon-β: a longitudinal study in multiple sclerosis patients. Clin Exp Immunol (2015) 179(1):119–27. doi: 10.1111/cei.12443 PMC4260904 25169051

[43] Izzo AA Sharkey KA. Cannabinoids and the gut: new developments and emerging concepts. Pharmacol Ther (2010) 126(1):21–38. doi: 10.1016/j.pharmthera.2009.12.005 20117132

[44] Lo Verme J Fu J Astarita G La Rana G Russo R Calignano A. The nuclear receptor peroxisome proliferator-activated receptor-alpha mediates the anti-inflammatory actions of palmitoylethanolamide. Mol Pharmacol (2005) 67(1):15–9. doi: 10.1124/mol.104.006353 15465922

[45] Solorzano C Zhu C Battista N Astarita G Lodola A Rivara S. Selective n-acylethanolamine-hydrolyzing acid amidase inhibition reveals a key role for endogenous palmitoylethanolamide in inflammation. Proc Natl Acad Sci USA (2009) 106(49):20966–71. doi: 10.1073/pnas.0907417106 PMC2791595 19926854

[46] Zhu C Solorzano C Sahar S Realini N Fung E Sassone-Corsi P. Proinflammatory stimuli control n-acylphosphatidylethanolamine-specific phospholipase d expression in macrophages. Mol Pharmacol (2011) 79(4):786–92. doi: 10.1124/mol.110.070201 PMC3063731 21233218

[47] Geurts L Everard A Van Hul M Essaghir A Duparc T Matamoros S. Adipose tissue NAPE-PLD controls fat mass development by altering the browning process and gut microbiota. Nat Commun (2015) 6:6495. doi: 10.1038/ncomms7495 25757720 PMC4382707

[48] Everard A Geurts L Caesar R Van Hul M Matamoros S Duparc T. Intestinal epithelial MyD88 is a sensor switching host metabolism towards obesity according to nutritional status. Nat Commun (2014) 5:5648. doi: 10.1038/ncomms6648 25476696 PMC4268705

[49] Bashashati M Storr MA Nikas SP Wood JT Godlewski G Liu J. Inhibiting fatty acid amide hydrolase normalizes endotoxin-induced enhanced gastrointestinal motility in mice. Br J Pharmacol (2012) 165(5):1556–71. doi: 10.1111/j.1476-5381.2011.01644.x PMC3372737 21883147

[50] Vanheel H Vicario M Vanuytsel T Van Oudenhove L Martinez C Keita ÅV. Impaired duodenal mucosal integrity and low-grade inflammation in functional dyspepsia. Gut (2014) 63(2):262–71. doi: 10.1136/gutjnl-2012-303857 23474421

[51] Hansen LBS Roager HM Søndertoft NB Gøbel RJ Kristensen M Vallès-Colomer M. A low-gluten diet induces changes in the intestinal microbiome of healthy Danish adults. Nat Commun (2018) 9(1):4630. doi: 10.1038/s41467-018-07019-x 30425247 PMC6234216

[52] Yousef MM Yantiss RK Baker SP Banner BF. Duodenal intraepithelial lymphocytes in inflammatory disorders of the esophagus and stomach. Clin Gastroenterol Hepatol (2006) 4(5):631–4. doi: 10.1016/j.cgh.2005.12.028 16630772

[53] Hahn HP Hornick JL. Immunoreactivity for CD25 in gastrointestinal mucosal mast cells is specific for systemic mastocytosis. Am J Surg Pathol (2007) 31(11):1669–76. doi: 10.1097/PAS.0b013e318078ce7a 18059223

[54] Zuberi RI Ge XN Jiang S Bahaie NS Kang BN Hosseinkhani RM. Deficiency of endothelial heparan sulfates attenuates allergic airway inflammation. J Immunol (2009) 183(6):3971–9. doi: 10.4049/jimmunol.0901604 PMC2872128 19710461

[55] Perreault N Katz JP Sackett SD Kaestner KH. Foxl1 controls the wnt/beta-catenin pathway by modulating the expression of proteoglycans in the gut. J Biol Chem (2001) 276(46):43328–33. doi: 10.1074/jbc.M104366200 11555641

[56] Berkhout M Gosens MJEM Brouwer KM Peters WHM Nagengast FM van Krieken JHJM. Loss of extracellular e-cadherin in the normal mucosa of duodenum and colon of patients with familial adenomatous polyposis. Hum Pathol (2006) 37(11):1389–99. doi: 10.1016/j.humpath.2006.05.018 16949915

[57] Zhang L Wei X Zhang R Si D Petitte JN Ahmad B. A novel peptide ameliorates LPS-induced intestinal inflammation and mucosal barrier damage *via* its antioxidant and antiendotoxin effects. Int J Mol Sci (2019) 20(16):3974. doi: 10.3390/ijms20163974 PMC6720008 31443263

[58] Caporaso JG Lauber CL Walters WA Berg-Lyons D Lozupone CA Turnbaugh PJ. Global patterns of 16S rRNA diversity at a depth of millions of sequences per sample. Proc Natl Acad Sci USA (2011) 108 Suppl 1:4516–22. doi: 10.1073/pnas.1000080107 PMC3063599 20534432

[59] Ji S Han S Yu L Du L You Y Chen J. Jia wei xiao yao San ameliorates chronic stress-induced depression-like behaviors in mice by regulating the gut microbiome and brain metabolome in relation to purine metabolism. Phytomedicine (2022) 98:153940. doi: 10.1016/j.phymed.2022.153940 35104765

