# Supplementary material for: Multi-omics analysis reveals the metabolic regulators of duodenal low-grade inflammation in a functional dyspepsia model
Source: Front Immunol. 2022 Aug 24;13:944591. doi: 10.3389/fimmu.2022.944591 (PMC9453867; doi:10.3389/fimmu.2022.944591)
Supplement: Supplementary file 2 [file DataSheet_1.docx]

Supplementary Material

# Supplementary Figures


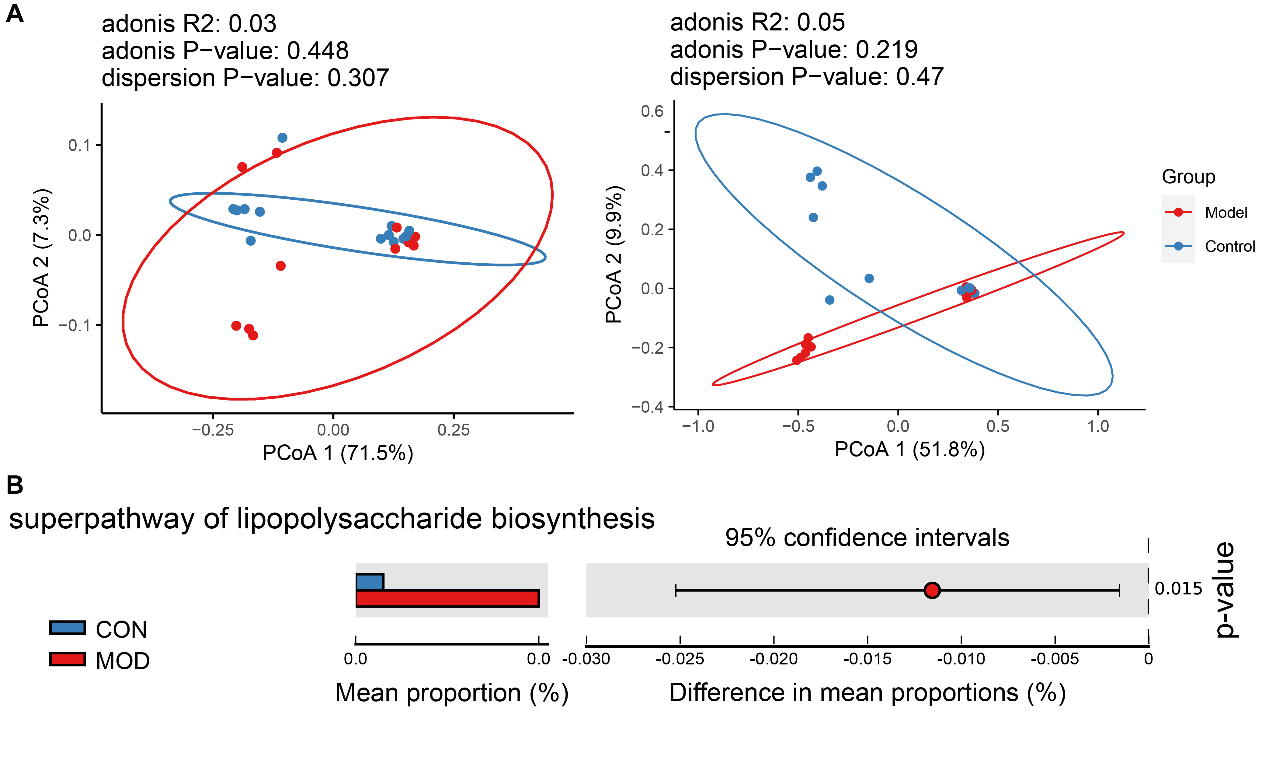


**Supplemental Figure 1. Pooled analysis of two external datasets (BioProject ID: PRJNA575916, PRJNA719295).** (**A**) Weighted UniFrac (left) and Bray-Curtis (right) with adonis tests. (**B**) Predicted MetaCyc pathway of fecal microbiome using PICRUSt2 analysis. Superpathway of lipopolysaccharide biosynthesis (LPSSYN-PWY) was enriched in the model group (*p* < 0.05 by Mann Whitney test) (control *n* = 11, model *n* = 15).


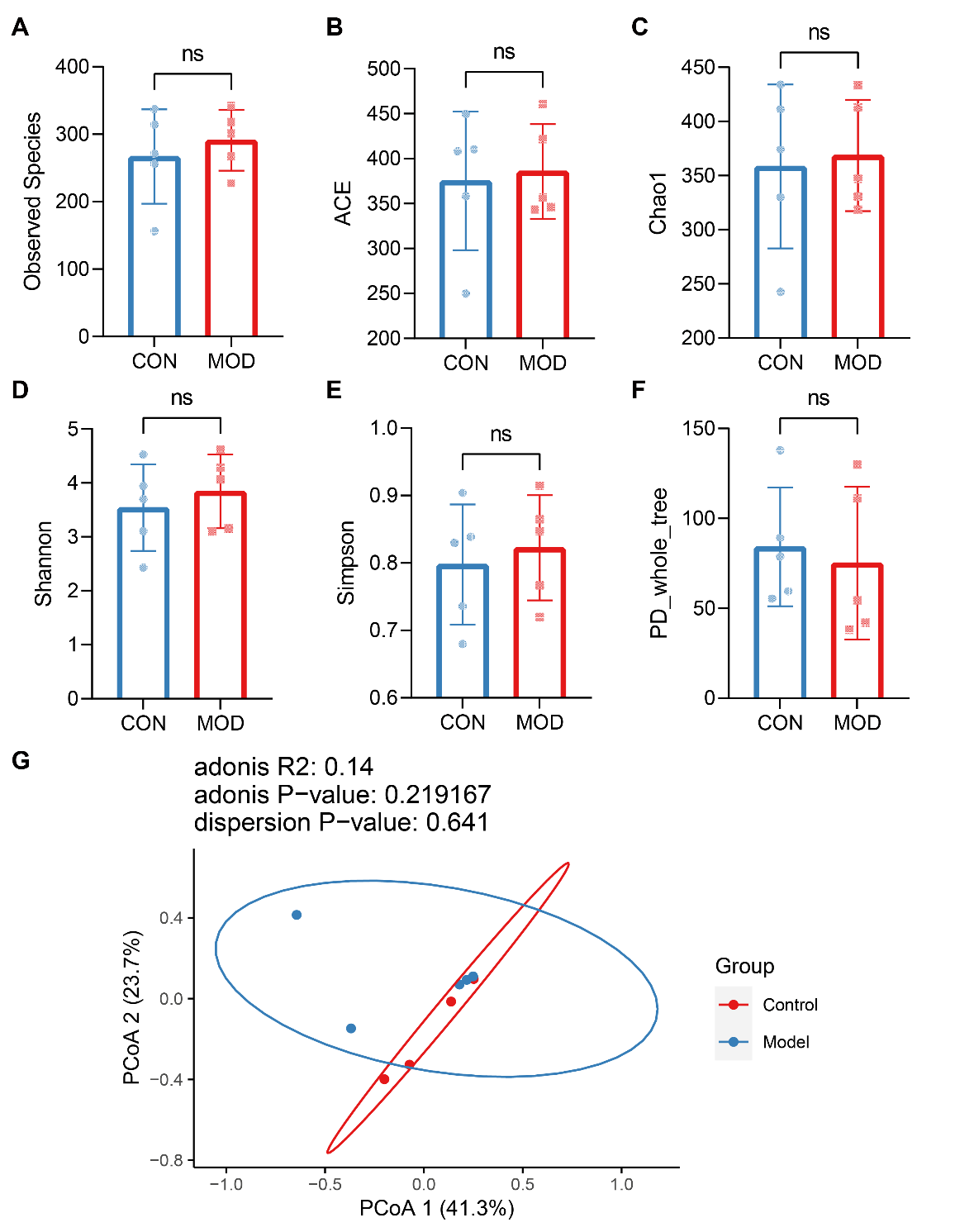


**Supplemental Figure 2. Alpha and beta diversity analysis of duodenal microbiome.** (**A-F**) All the alpha diversity including observed species (**A**), ACE (**B**), Chao1 (**C**), Shannon (**D**), Simpson (**E**) and PD_whole_tree (**F**) indicated no significant (unpaired t test with Welch’s correction). (**G**) Dissimilarity between samples was calculated by Bray-Curtis with an adonis test, indicating no significant (*n* = 5 rats/group).


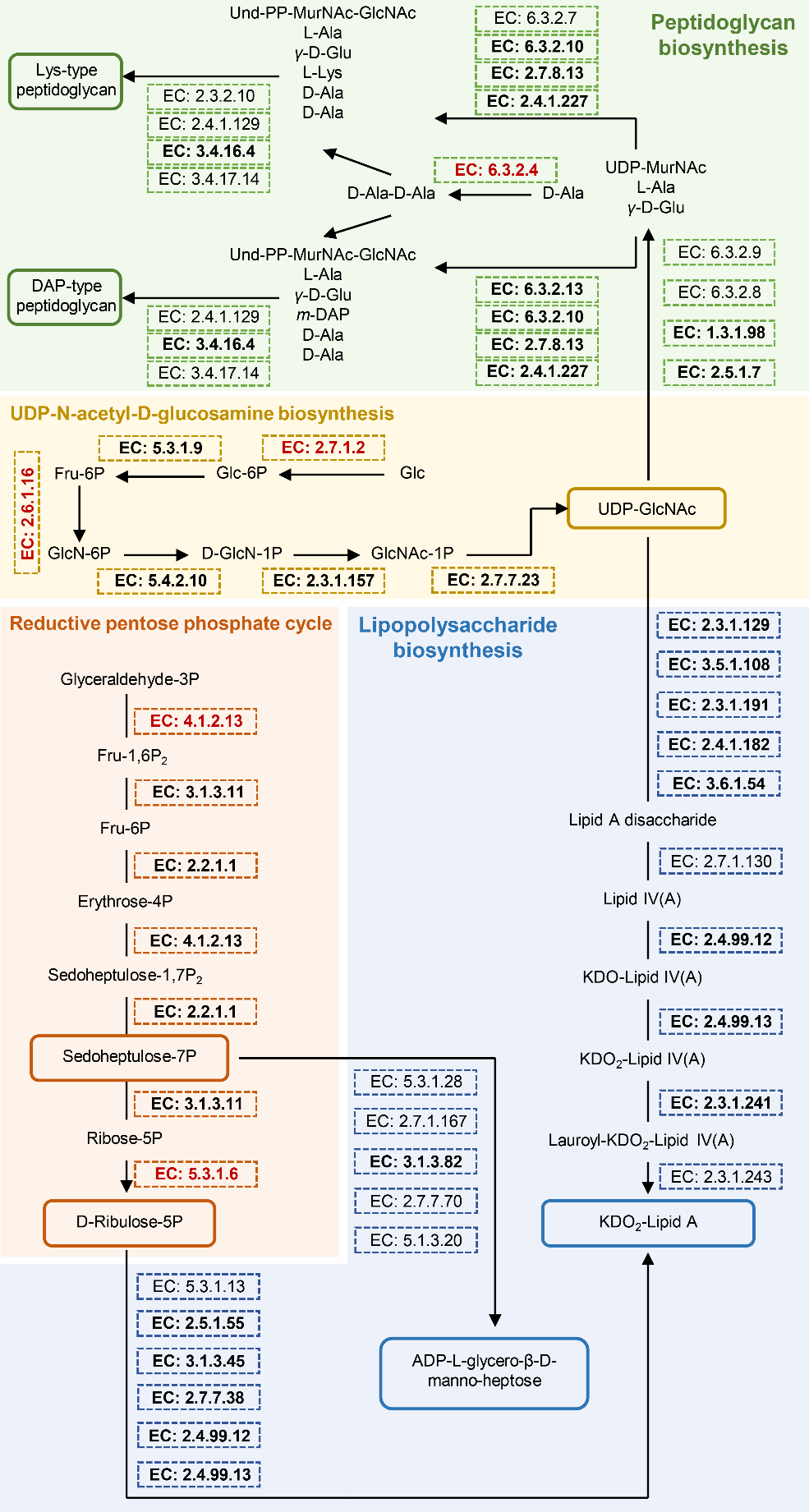


**Supplemental Figure 3. Predicted metabolic enzymes involved in the biosynthesis of lipopolysaccharide and peptidoglycan of duodenal microbiome.** Differential enzymes were marked with dark red (two-way ANOVA with pairwise comparison of Fisher’s LSD, *p* < 0.05 represent significant). The majority of enzymes trending higher in model group were bolded, although not significantly different.


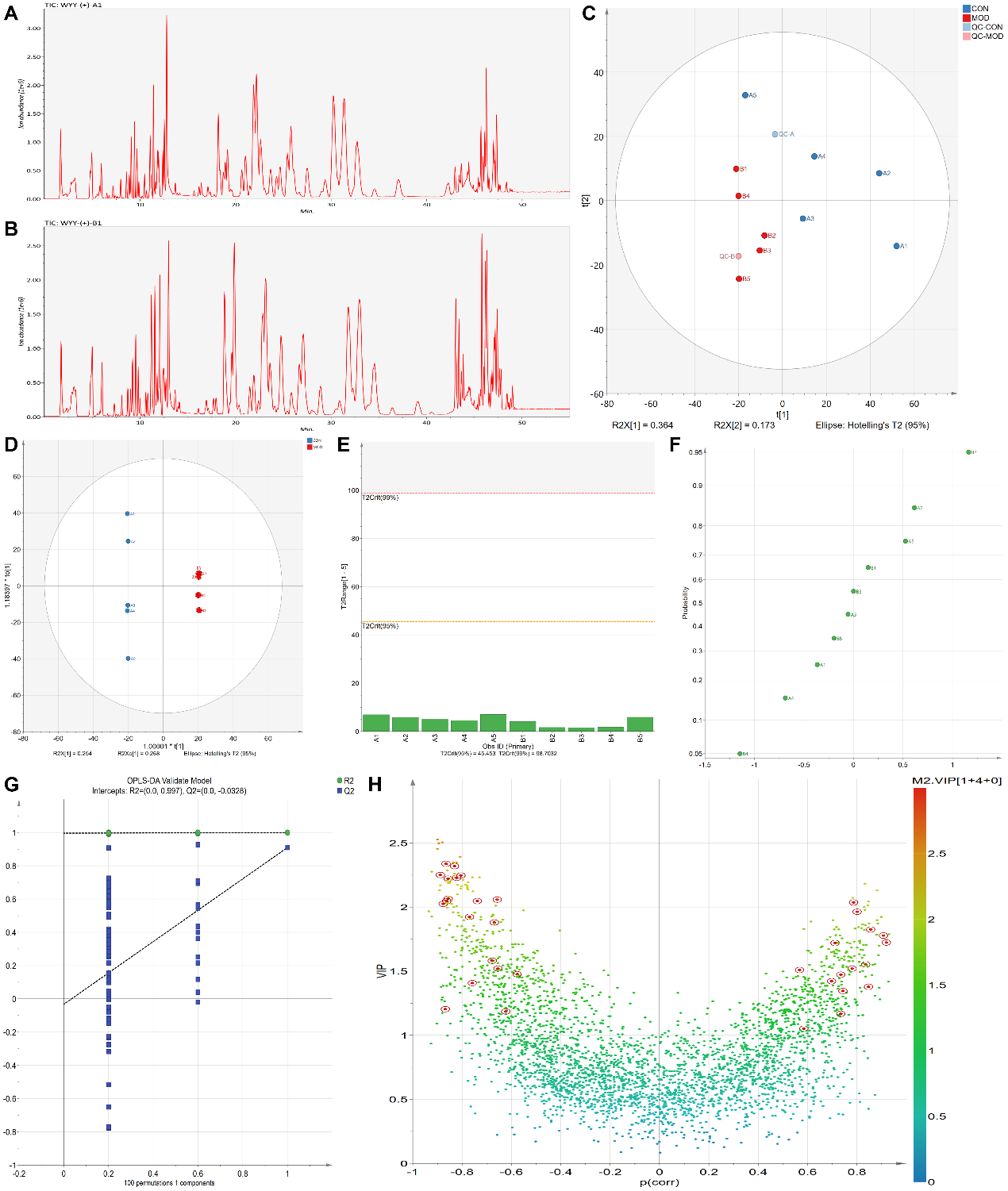


**Supplemental Figure 4. Data processing in positive mode.** (**A**) Representative chromatogram of the control group. (**B**) Representative chromatogram of the model group. (**C**) PCA analysis of all samples with QC. (**D**) OPLS-DA analysis of all samples. (**E-G**) Model validation. Hotelling's T2 (**E**), Residuals Normal Probability (**F**) and Permutation tests (**G**) were used to evaluate the model. (**H**) Primary screening metabolites were red-labeled in a volcano plot.


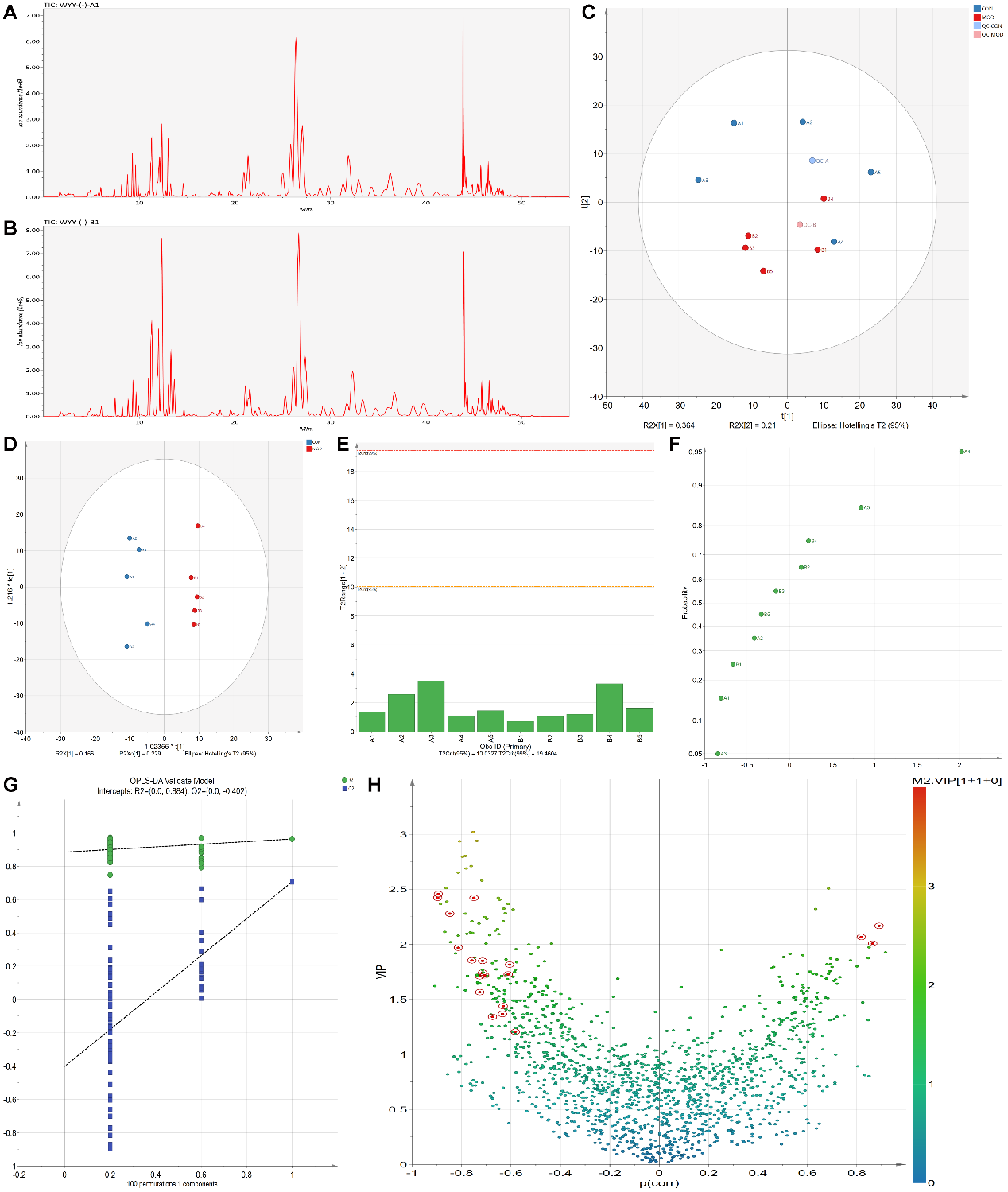


**Supplemental Figure 5.** **Data processing in negative mode.** (**A**) Representative chromatogram of the control group. (**B**) Representative chromatogram of the model group. (**C**) PCA analysis of all samples with QC. (**D**) OPLS-DA analysis of all samples. (**E-G**) Model validation. Hotelling's T2 (**E**), Residuals Normal Probability (**F**) and Permutation tests (**G**) were used to evaluate the model. (**H**) Primary screening metabolites were red-labeled in a volcano plot.


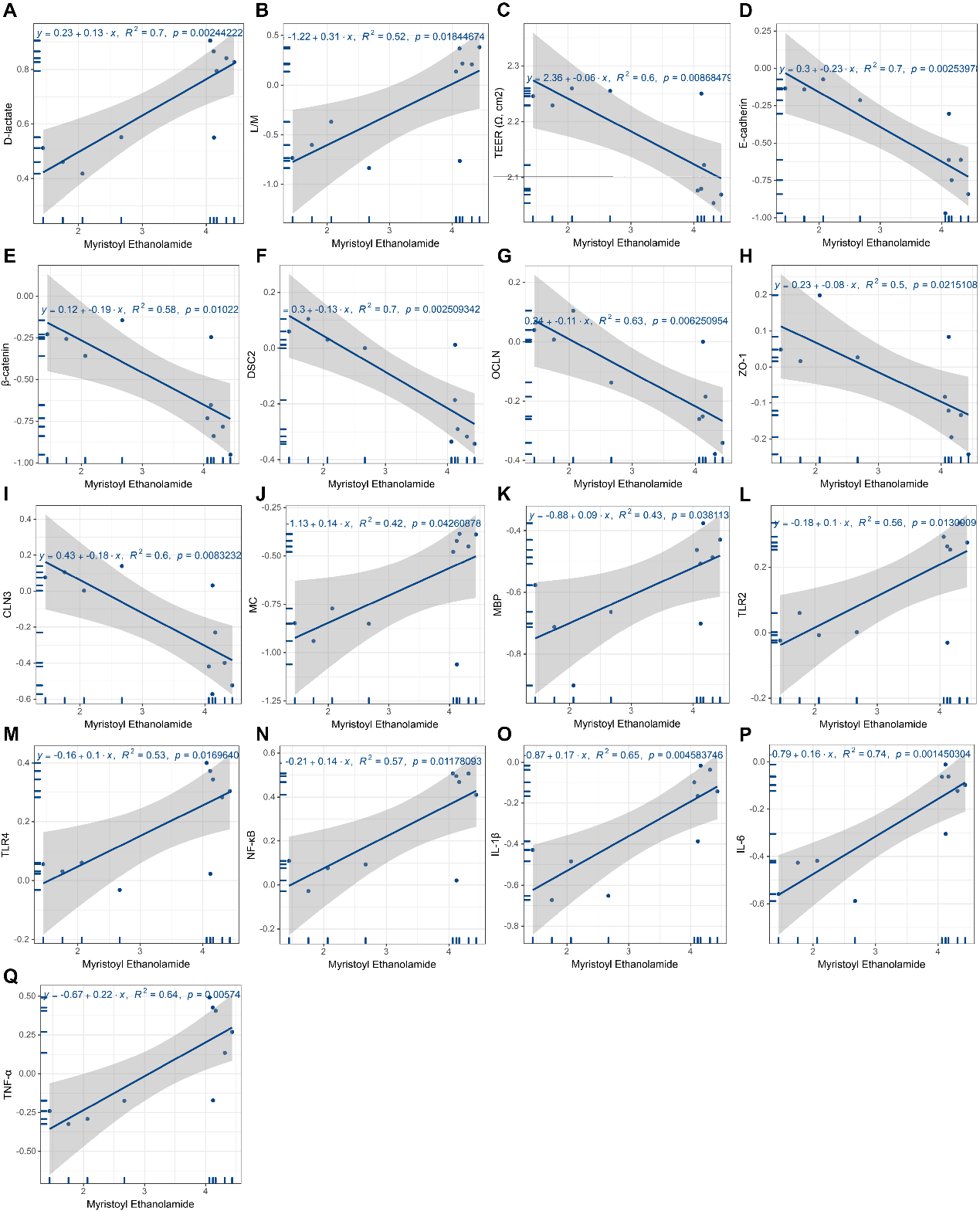


**Supplemental Figure** **6.** **Linear regression analysis of MEA and features of FD.** (**A-C**) Duodenal permeability and absorption. MEA was positively linear correlated with plasma content of D-lactate (**A**), L/M ratio (**B**), while negatively linear correlated with TEER (**C**). (**D-I**) Duodenal mucosal barrier. MEA was negatively linear corelated with the relative protein expressions of E-cadherin (**D**) and β-catenin (**E**) and the relative mRNA expressions of *Dsc2* (**G**), *Ocln* (**H**) and *Cln3* (**I**). (**J-Q**) Innate immune cells and proinflammatory signaling. MEA was positively linear correlated with the relative amounts of mast cells (**J**), eosinophils (**K**), the relative mRNA expressions of *Tlr2* (**L**), *Tlr4* (**M**) and *Rela* (**N**), and the contents of proinflammatory cytokines IL-1β (**O**), IL-6 (**P**) and TNF-α (**Q**) (All data are log10 transformed. *p* < 0.05 represent significant) (n = 5 rats/group).


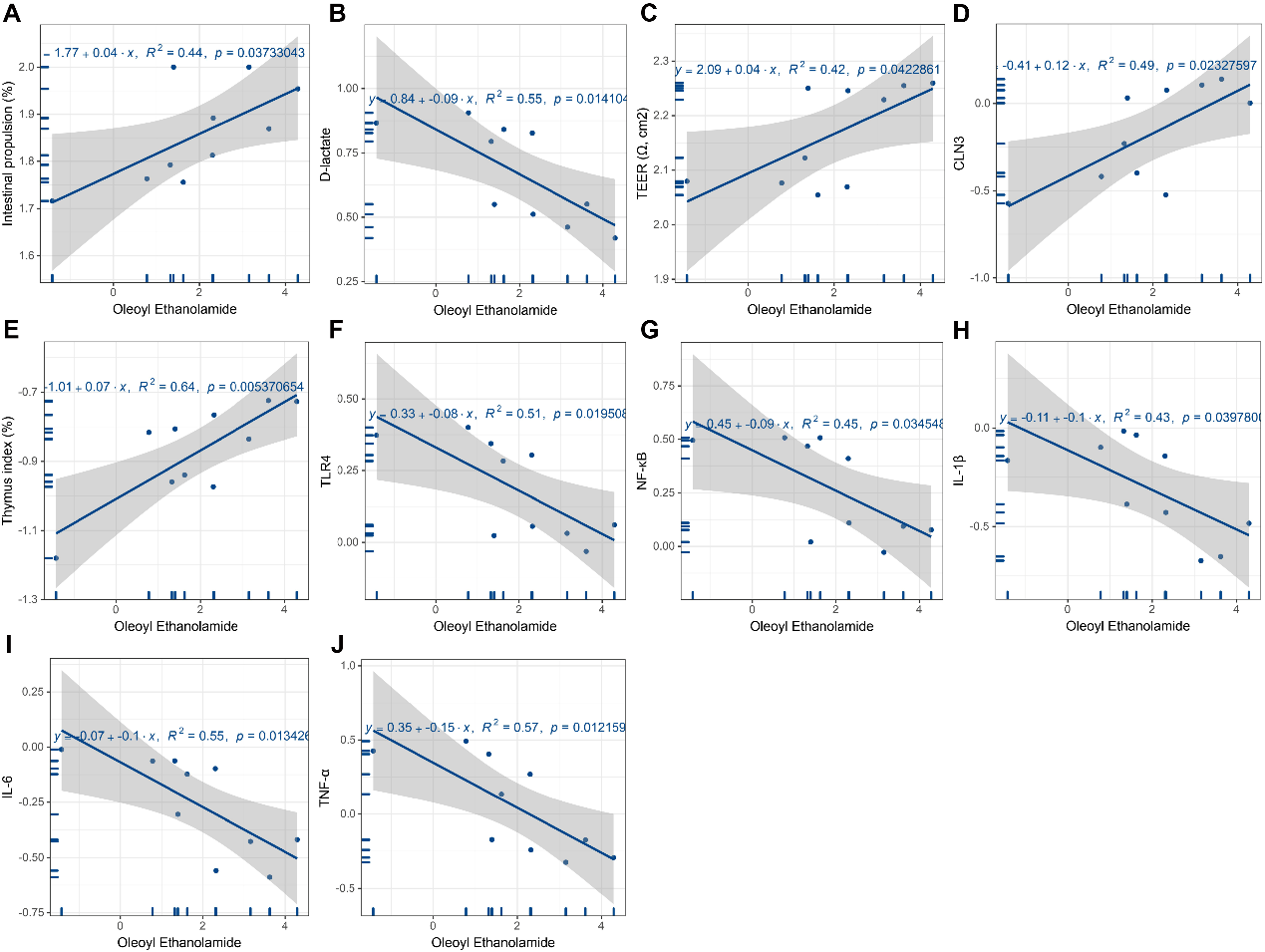


**Supplemental Figure 7. Linear regression analysis of OEA and features of FD.** (**A**) Gastrointestinal motor function. OEA was positively linear correlated with the gastrointestinal transit rate. (**B-C**) Duodenal permeability and absorption. OEA was negatively linear correlated with plasma content of D-lactate (**B**) while positively linear correlated with TEER (**C**). (**D**) Duodenal mucosal barrier. OEA was positively linear corelated with the relative mRNA expressions of *Cln3*. (**E**) Immune organ index. OEA was positively linear corelated with thymus index. (**F-J**) Proinflammatory signaling. OEA was negatively linear correlated with the relative mRNA expressions of *Tlr4* (**F**) and *Rela* (**G**), and the contents of proinflammatory cytokines IL-1β (**H**), IL-6 (**I**) and TNF-α (**J**) (All data are log10 transformed. *p* < 0.05 represent significant) (n = 5 rats/group).


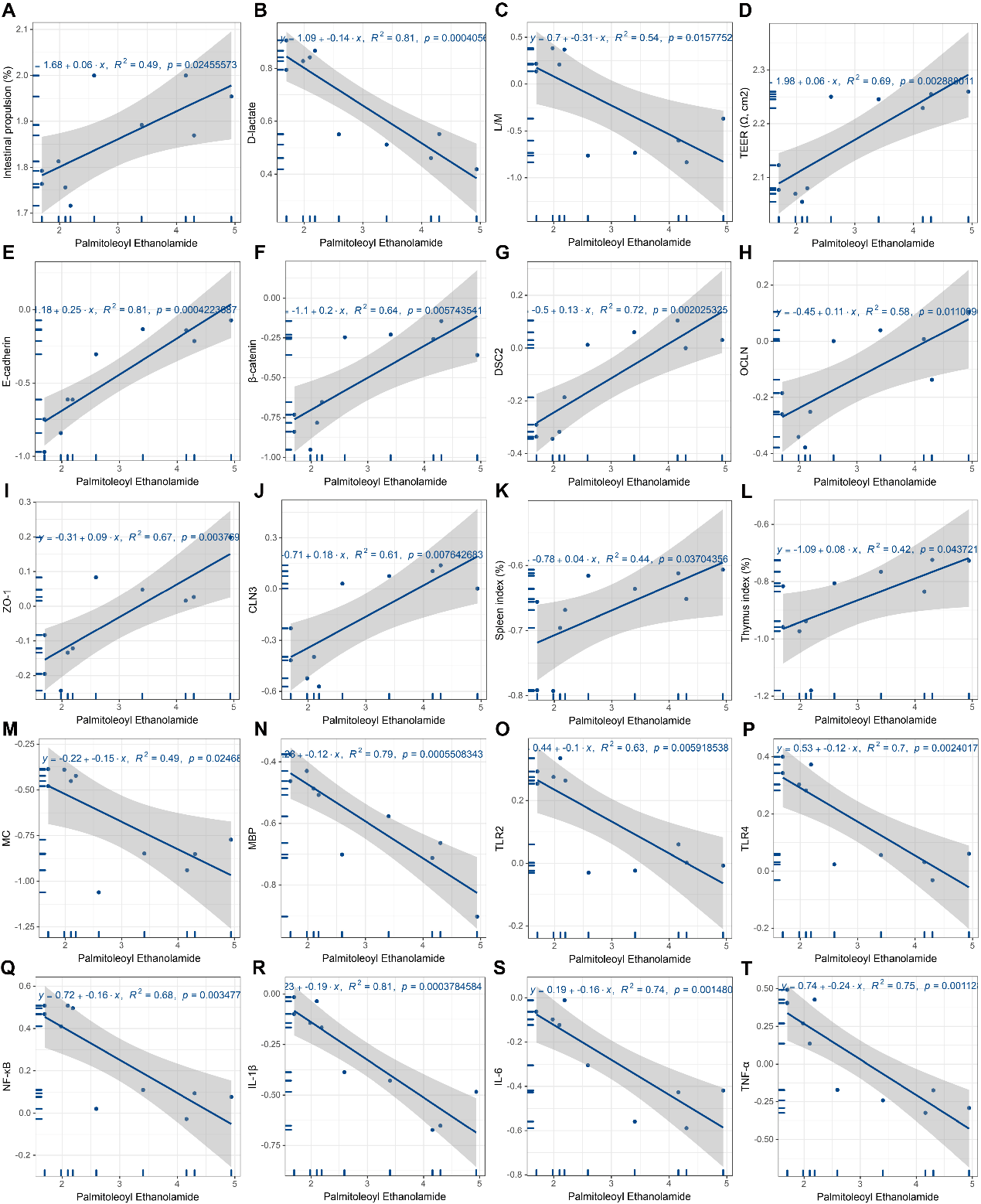


**Supplemental Figure 8. Linear regression analysis of PEA and features of FD.** (**A**) Gastrointestinal motor function. PEA was positively linear correlated with the gastrointestinal transit rate. (**B-D**) Duodenal permeability and absorption. PEA was negatively linear correlated with plasma content of D-lactate (**B**) and L/M ratio (**C**), while positively linear correlated with TEER (**D**). (**E-J**) Duodenal mucosal barrier. PEA was positively linear corelated with the relative protein expressions of E-cadherin (**E**) and β-catenin (**F**) and the relative mRNA expressions of *Dsc2* (**G**), *Ocln* (**H**), *Tjp1* (**I**) and *Cln3* (**J**). (**K, L**) Immune organ index. PEA was positively linear corelated with spleen (**K**) and thymus (**L**) index. (**M-T**) Innate immune cells and proinflammatory signaling. PEA was negatively linear correlated with the relative amounts of mast cells (**M**), eosinophils (**N**), the relative mRNA expressions of *Tlr2* (**O**), *Tlr4* (**P**) and *Rela* (**Q**), and the contents of proinflammatory cytokines IL-1β (**R**), IL-6 (**S**) and TNF-α (**T**) (All data are log10 transformed. *p* < 0.05 represent significant) (n = 5 rats/group).


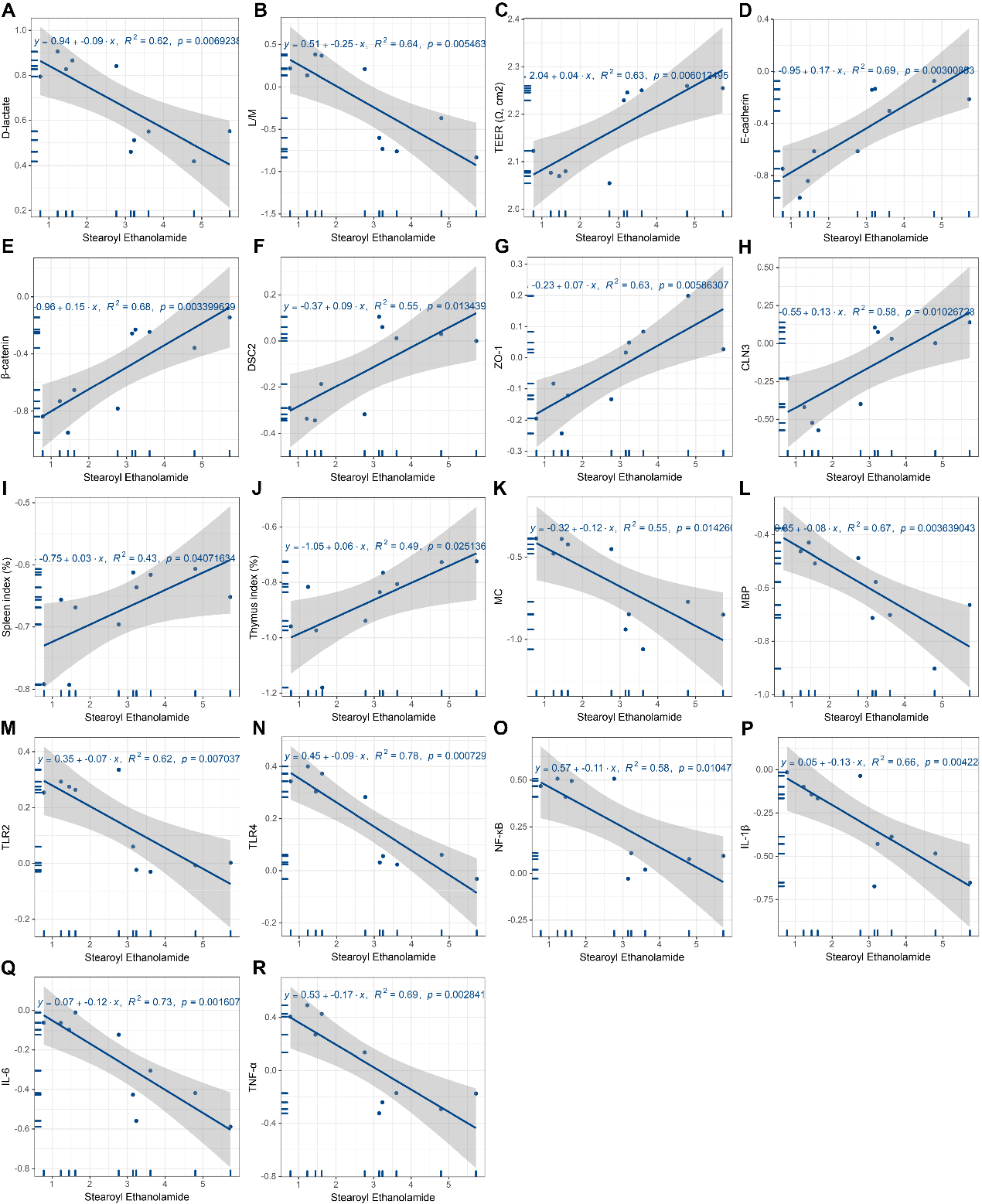


**Supplemental Figure 9. Linear regression analysis of SEA and features of FD.** (**A-C**) Duodenal permeability and absorption. SEA was negatively linear correlated with plasma content of D-lactate (**A**) and L/M ratio (**B**), while positively linear correlated with TEER (**C**). (**D-H**) Duodenal mucosal barrier. SEA was positively linear corelated with the relative protein expressions of E-cadherin (**D**) and β-catenin (**E**) and the relative mRNA expressions of *Dsc2* (**F**), Tjp1 (**G**) and *Cln3* (**H**). (**I, J**) Immune organ index. SEA was positively linear corelated with spleen (**I**) and thymus (**J**) index. (**K-R**) Innate immune cells and proinflammatory signaling. SEA was negatively linear correlated with the relative amounts of mast cells (**K**), eosinophils (**L**), the relative mRNA expressions of *Tlr2* (**M**), *Tlr4* (**N**) and *Rela* (**O**), and the contents of proinflammatory cytokines IL-1β (**P**), IL-6 (**Q**) and TNF-α (**R**) (All data are log10 transformed. *p* < 0.05 represent significant) (n = 5 rats/group).
